# Indole Derivatives Obtained from Egyptian *Enterobacter* sp. Soil Isolates Exhibit Antivirulence Activities against Uropathogenic *Proteus mirabilis*

**DOI:** 10.3390/antibiotics10040363

**Published:** 2021-03-29

**Authors:** Mai A. Amer, Reham Wasfi, Ahmed S. Attia, Mohamed A. Ramadan

**Affiliations:** 1 Department of Microbiology and Immunology, Faculty of Pharmacy, October University for Modern Sciences and Arts (MSA), Giza 12451, Egypt; mwahed@msa.edu.eg (M.A.A.); rwasfi@msa.edu.eg (R.W.); 2 Department of Microbiology and Immunology, Faculty of Pharmacy, Cairo University, Cairo 11562, Egypt; Mohamed.abdelhalim@pharma.cu.edu.eg; 3 Department of Microbiology and Immunology, School of Pharmacy, Newgiza University, Giza 11341, Egypt

**Keywords:** *Proteus mirabilis*, microbial indole derivatives, anti-urease, anti-biofilm, anti-swarming, *Enterobacter* sp.

## Abstract

*Proteus mirabilis* is a frequent cause of catheter associated urinary tract infections (CAUTIs). Several virulence factors contribute to its pathogenesis, but swarming motility, biofilm formation, and urease activity are considered the hallmarks. The increased prevalence in antibiotic resistance among uropathogens is alarming and requires searching for new treatment alternatives. With this in mind, our study aims to investigate antivirulence activity of indole derivatives against multidrug resistant *P. mirabilis* isolates. Ethyl acetate (EtOAc) extracts from *Enterobacter* sp. (rhizobacterium), isolated from Egyptian soil samples were tested for their ability to antagonize the virulence capacity and biofilm activity of *P. mirabilis* uropathogens. Extracts of two *Enterobacter* sp. isolates (coded Zch127 and Cbg70) showed the highest antivirulence activities against *P. mirabilis*. The two promising rhizobacteria Zch127 and Cbg70 were isolated from soil surrounding: *Cucurbita pepo* (Zucchini) and *Brassica oleracea var. capitata L.* (Cabbage), respectively. Sub-minimum inhibitory concentrations (Sub-MICs) of the two extracts showed potent antibiofilm activity with significant biofilm reduction of ten *P. mirabilis* clinical isolates (*p*-value < 0.05) in a dose-dependent manner. Interestingly, the Zch127 extract showed anti-urease, anti-swarming and anti-swimming activity against the tested strains. Indole derivatives identified represented key components of indole pyruvate, indole acetamide pathways; involved in the synthesis of indole acetic acid. Additional compounds for indole acetonitrile pathway were detected in the Zch127 extract which showed higher antivirulence activity. Accordingly, the findings of the current study model the feasibility of using these extracts as promising antivirulence agent against the *P. mirabilis* uropathogens and as potential therapy for treatment of urinary tract infections (UTIs).

## 1. Introduction

Urinary tract infections (UTI) are the major cause of healthcare associated infections accounting for 40% of all hospital acquired infections. Catheter associated urinary tract infections (CAUTI) represent approximately 80% of Healthcare associated UTIs [1]. Bacteria that belong to the family Enterobacteriaceae are the most frequent causative agent of UTIs. Clinically, *Proteus mirabilis* is the most common cause of complicated urinary tract infections, particularly in patients suffering from long-term catherization. These infections are complicated by catheter encrustation and blockage resulting from the ability of this bacterium to produce crystalline biofilm [2].

*P. mirabilis* is an opportunistic pathogen, which is widely spread in the environment, mainly found in water, soil, and the gastrointestinal tracts of humans and animals. *P. mirabilis* uses a diverse set of virulence factors to access and colonize the host urinary tract. These virulence factors include flagella, fimbriae, urease enzyme, capsule polysaccharide, and efflux pump resulting in crystalline biofilm formation [3]. *P. mirabilis* UTIs are often very persistent and difficult to be treated due to its intrinsic resistance to polymyxin, tetracycline, and nitrofurazone [2], as well as the emergence of multidrug resistant strains [4]. CAUTI formed by *P. mirabilis* are difficult to be treated because of their ability to form dense crystalline biofilm which offers protection for the bacterial cells within the matrix from antibiotics and immune system [3]. *P. mirabilis* has been shown to enter the viable but non culturable state (VBNC) under conditions of high osmotic pressure and yet retaining virulence [5]. All the previously mentioned reasons made searching for new alternatives for antimicrobial agents crucial.

Antivirulence compounds attract increasing interest recently because they affect bacterial virulence without creating selection pressure on the microorganisms and thus reduce the development of antibiotic resistance. Inhibiting virulence factors could attenuate bacterium pathogenicity, and consequently enable the host immune system to successfully combat the pathogen [6]. In the recent years, indole has been gaining much attention as an intercellular, interspecies, and interkingdom signaling molecule [7]. Several studies have shown that some indole derivatives can inhibit the virulence activity of several bacteria including *Escherichia coli* O157:H7, *Pseudomonas aeruginosa*, *Salmonella enterica* serovar Typhimurium, and *Staphylococcus aureus* [6,8,9,10,11,12,13]. Another study carried out by Palaniyandi and co-workers reported that phytohormones metabolites produced by rhizobacteria was found to protect host plant against pathogens [14]. Thus, the aim of this study was exploring the antibiofilm and antivirulence activities of natural metabolites derived from indole, produced by rhizobacteria, against clinical isolates of *P. mirabilis* and up to the best of our knowledge this is the first report on the antivirulence effect of these metabolites on *P. mirabilis*.

## 2. Results and Discussion

### 2.1. Some Rhizobacteria Enterobacter sp. Isolates Exhibit High Indole Production Coupled with Potential Antivirulence Activity against P. mirabilis

Out of twelve collected soil samples from different locations in Egypt, a total of 137 bacterial isolates were collected. Sixty isolates were indole producer as detected by the positive color reaction with Salkowski’s reagent and among them 13 isolates showed highest indole production (Table S1). Ethyl acetate extracts of the highest indole producing isolates were tested for their antivirulence activity against *P. mirabilis* (P1 isolate). Competition between microorganisms living in the environment drives them to produce a wide range of secondary metabolites to aid their survival in the presence of other microbes [7], hence they become a source for potential antivirulent agents. 

**Figure 1:**
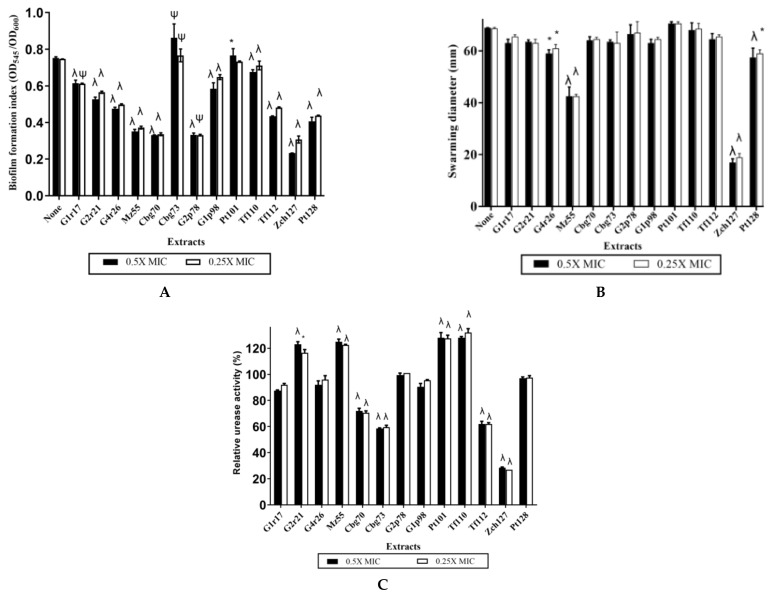
Antivirulence activity of 13 bacterial culture extracts on *P. mirabilis* P1 isolate. The effect of 0.5X of minimum inhibitory concentration (MIC) (Extracts no. G1r21, Cbg73, G2p78: 1.25 mg/mL, G1r17, G1p98, Zch127: 0.625 mg/mL extracts no. Mz55, Tf110, Tf112, Pt128: 0.312 mg/mL, extracts no. Pt101: 0.156 mg/mL and extracts G4r26, Cbg70: 0.08 mg/mL) and 0.25X MIC of the bacterial culture extracts containing indole derivatives on *P. mirabilis* isolate P1 (**A**) Normalized biofilm formation, (**B**) Diameter of swarming motility, and (**C**) Relative urease activity, where the values were obtained in the absence of extracts after 3 h of incubation were set as 100 %, and all other values were expressed relative to this value. Data represents the mean of at least 3 biological replicas. Statistical analysis using two-way ANOVA, which was followed by multiple comparisons test with a significance level at * *p* < 0.05; ^ψ^ *p* < 0.01; ^λ^ *p* < 0.001.

Two rhizobacterial isolates, coded as “Cbg70” and “Zch127”, were selected for further study due to their potent antivirulence activity, including antibiofilm, anti -swarming and anti-urease activities, against *P. mirabilis* isolate (P1) compared to other tested extracts (Figure 1). The “Cbg70” and “Zch127” were isolated from soil surrounding the roots of *Brassica oleracea var. capitata L.* (Cabbage) and *Cucurbita pepo* (Zucchini), respectively. Cruciferous (or *Brassica*) vegetables, including cauliflower, brussels, broccoli, and cabbage, are rich sources for indole derivatives [15,16], and previous studies have revealed that indole-3-carbinol and 3,3′-bismethyl indole from cruciferous vegetables have antiviral, antimicrobial and anticancer activities [16,17]. Indole derivatives derived from natural sources such as cruciferous plants and actinomycetes as *Rhodococcus* sp. BFI 332 were reported to have antibiofilm activity against *Escherichia coli, Staphylococcus aureus*, and *Pseudomonas aeruginosa* [9,10,12].

Isolates of Cbg70 and Zch127 were identified by 16s *rRNA* gene sequencing, up to the genus level, as *Enterobacter* sp. *Enterobacter* sp. has been previously shown to be abundant in the soil samples [18,19,20]. The phylogenetic tree of the two selected strains and highly similar strains in the NCBI database was constructed using Mega 6.0 (Figure S1). The two isolated strains were found to belong to the same clade and most of the phylogenetically related strains to these isolates were previously recovered from soil samples as reported in the NCBI database. 

### 2.2. Half MICs of Cbg70 and Zch127 Have no Inhibitory Effect on Growth of P. mirabilis

**Figure 2:**
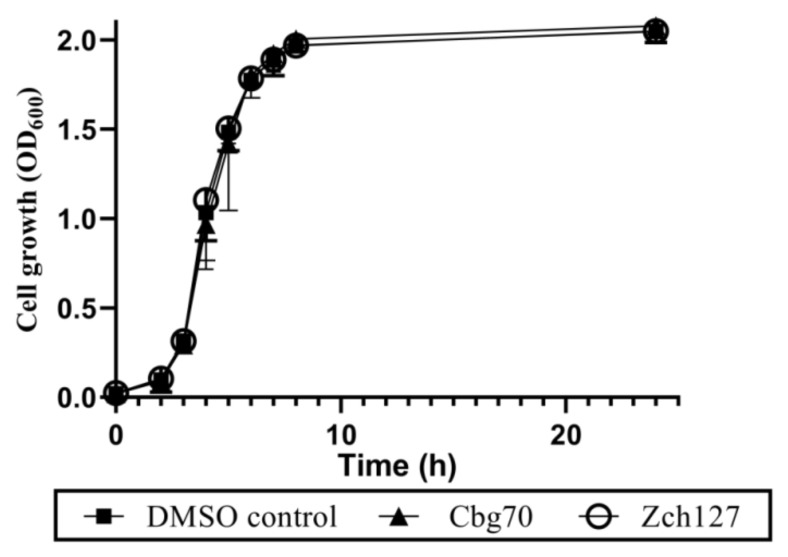
Effect of *Enterobacter* sp. culture supernatant extracts on bacterial growth of *P. mirabilis*. Growth of *Proteus mirabilis* isolates under the effect of sub-minimum inhibitory concentrations (sub-MIC) (0.5X MIC) concentrations of Cbg70 (0.08 mg/mL) and Zch127 (0.6 mg/mL) extracts at different time points measured as turbidity (OD_600_ nm) while shaking at 250 rpm. Bacterial culture supplemented with DMSO in the same concentration as extract served as DMSO control. Each experiment was performed using three different cultures for each isolate, and the average data of the ten isolates is shown.

The minimum inhibitory concentrations (MICs) of the Cbg70 and Zch127 culture supernatant extracts against the ten multidrug resistant *P. mirabilis* isolates were 0.16 and 1.25 mg/mL, respectively. The effect of half MIC of extracts on the growth of *P. mirabilis* isolates showed no significant (*p* > 0.05) inhibitory effect compared with the control using two-way ANOVA followed by multiple comparisons test (Figure S2 and S3). The average of cell growth of the *P. mirabilis* isolates in the presence of half MIC of extracts at different time points were shown in Figure 2.

### 2.3. Sub MIC Concentrations of Cbg70 and Zch127 Extracts Significantly Inhibit P. mirabilis Biofilm Formation

**Figure 3:**
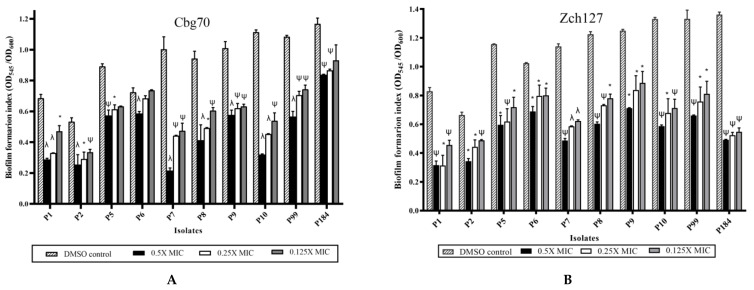
Inhibitory effect of *Enterobacter* sp. culture supernatant on biofilm formation of *P. mirabilis* isolates. Average biofilm formation index of *P. mirabilis* isolates following treatment with sub- MICs of the indole extracts: DMSO control (striped bars), at 0.5X MIC (black bars), 0.25X MIC (white bars), and 0.125X MIC (grey bars): (**A**) Culture supernatant of extract Cbg70 at concentrations ranging from (20 to 80 µg/mL), (**B**) Culture supernatant of extract Zch127 at concentrations ranging from (0.15 to 0.6 mg/mL)**,** Data represents the mean of at least 3 biological replicates, and error bars show standard error of the mean. Statistically difference was determined by student’s *t*-test * *p* < 0.05; ^ψ^ *p* < 0.01; ^λ^ *p* < 0.001 compared with DMSO control.

Tested *P. mirabilis* isolates showed variable biofilm forming ability, where 70% of isolates were strong and 30% were moderate biofilm forming. The indole extract from the selected *Enterobacter* strains showed antibiofilm activity against *P. mirabilis* reaching up to 65–80% reduction and the effect was concentration dependent. Significant reduction (*p*-value < 0.05) in biofilm biomass was observed among all the treated *P. mirabilis* isolates using 0.5X MIC of the Cbg70 and Zch127 extracts (Figure 3). Similar reduction percentage was observed by Lee et al. [10] using actinomycete *Rhodococcus* sp. BFI 332 indole containing extracts that inhibited biofilm formation in *E. coli* O157:H7 by 70%. The antibiofilm effect of indole derivatives could be attributed to their effect in decreasing curli production [9] or their role in signaling between bacterial cells at the intra and inter species levels [8].

### 2.4. Indole Extract Zch 127 Inhibited the Swimming and Swarming Motility of P. mirabilis 

**Figure 4:**
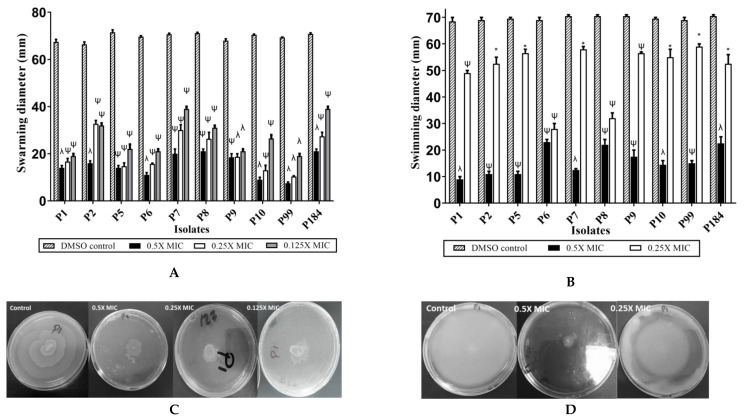
Inhibitory Effect of Zch127 culture extract on motility. The effect of Zch127 at sub-MICs on the motility of 10 *P. mirabilis* isolates (**A**) Swarming motility at 0.125X, 0.25X, and 0.5X MIC; (**B**) Swimming motility at 0.25X and 0.5X MIC, Data represents the mean of 3 experiments, and error bars shows standard error. Statistical difference was determined by student’s *t*-test, * *p* < 0.05; ^ψ^ *p* < 0.01; ^λ^ *p* < 0.001 compared with DMSO control; (**C**) Representative images of the effect of extract Zch127 on swarming motility of *P. mirabilis* isolate P1; (**D**) Representative images of the effect of extract Zch127 on swimming motility of *P. mirabilis* isolate P1.

Sub-minimum inhibitory concentrations (Sub-MICs) of the crude extract “Zch127” showed inhibitory effect on the swarming and swimming motilities of the *P. mirabilis* isolates without any effect on growth (Figure 4). The anti-swarming and anti-swimming effects were concentration dependent (Figures S4–S6), while Cbg70 extract showed no effect on bacterial motility (data not shown). Flagellar-mediated swimming motility is associated with biofilm formation because it enables the bacteria to reach substratum and start the attachment to surface [21]. Studies showed that antibiofilm activity of some natural compounds is coupled with its ability to inhibit the swarming differentiation [22,23].

### 2.5. Inhibitory Effect of Zch127 Crude Extract on the Urease Activity of P. mirabilis 

**Figure 5:**
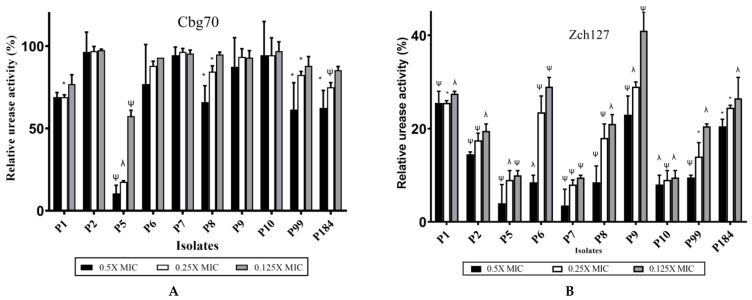
Effect of crude supernatant extracts of Cbg70 and Zch127 on urease activity. The relative urease activity of the *P. mirabilis* isolates in the presence of sub-MICs of extracts: (**A**) Cbg70; and (**B**) Zch127, at different concentrations (0.5X, 0.25X and 0.125X MIC). The values were obtained in the absence of extract after 3 h of incubation were set at 100 %, and all other values were expressed relative to this value. The data represents the means of three independent experiments and error bars show standard error of the mean. Statistical difference was determined by student’s *t*-test * *p* < 0.05; ^ψ^ *p* < 0.01; ^λ^ *p* < 0.001 compared with DMSO control.

In Christensen’s medium, untreated *P. mirabilis* was able to utilize the urea as the sole source of nitrogen, thus producing an amount of ammonia sufficient to change the color of the pH indicator (phenol red) in the medium and that was measured spectrophotometrically after 3 h of incubation. The Cbg70 extract showed variable effect on the urease activity of the tested *P. mirabilis* isolates. Significant reduction (*p* < 0.05) in urease activity was observed in 4 isolates, only namely P5, P8, P99, and P184 by 90%, 34%, 39%, and 38%, respectively, compared to untreated control (Figure 5A). Treatment of *P. mirabilis* isolates with the extract Zch127 significantly (*p* < 0.05) reduced the urease activity after 3 h of incubation in all isolates and that effect was concentration dependent (Figure 5B).

### 2.6. The Effect of Cbg70 and Zch127 Crude Extracts on the Antibiotic Sensitivity of P. mirabilis 

The effect of combining the extracts Cbg70 and Zch127, at sub-MICs, was assayed with three antibiotics: ceftriaxone, ciprofloxacin, and amikacin. The extract Zch127 at sub-MIC showed synergistic activity with the antibiotics: ceftriaxone and amikacin with most of the tested isolates. The same extract showed antagonistic effect with ciprofloxacin against 40% (4/10) of isolates (Table 1). The effect of the extract Cbg 70 was variable with different antibiotics with the majority of these effects were antagonistic to the effect of antibiotic (Table 1). Some small molecules have been previously reported to cause synergistic activity with antibiotics against pathogenic bacteria [24]. Previous studies reported change in antibiotic resistance in presence of indole and indole derivatives with different microorganisms. The change was variable according to the bacterial strain, indole derivatives [6,25,26], and the mechanism underlying antibiotic resistance [27].

### 2.7. Identification of Indole Derivatives in the Crude Extracts by LC-MS Analysis

LC-MS was employed to identify the bioactive compounds in the ethyl acetate (EtOAc) fraction of extracts Cbg70 and Zch127 after four days of incubation. The analysis was carried out in the positive and the negative modes (Figure S7). Compounds were identified based on their molecular ion mass, mass fragmentation pattern, and absorption spectra. As shown in Table 2 and Figures S8–S11, the described spectrum was indicative for the presence of eight indole derivatives in the extract Cbg70 where indole acetic acid (IAA), indole-3-ethanol (TOL), and indole-3-acetaldehyde were detected in both modes, tryptophan (Trp), indole-3-pyruvic acid (IPA) and indole-3-aldehyde were detected in the positive mode while indole-3-propionic acid and indole-3-acetamide were detected in the negative mode. Indole derivatives in Zch127 extract were ten, where IAA, TOL, IPA and 3-methyl indole were detected in both modes, Trp and indole-3-acetamide were detected in the negative mode, while indole-3-lactic acid, indole-3-acetaldehyde, indole-3-carboxylic acid, and indole-3-acetonitrile were detected in the positive mode. 

### 2.8. Identification and Verification of Indole Compounds in the Extracts Using HPLC

**Figure 6:**
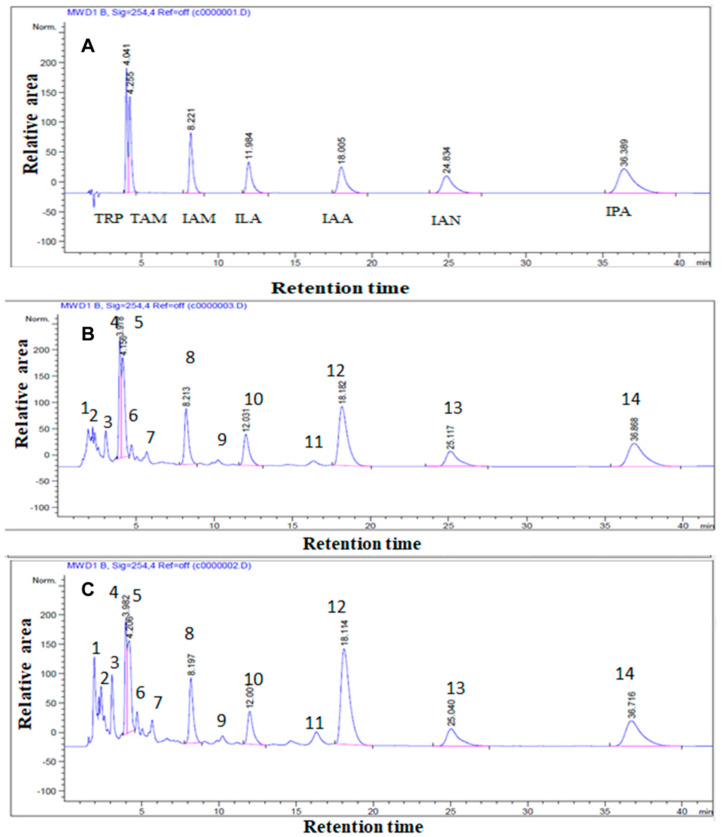
HPLC chromatograms of the authentic indole compounds (**A**) and the ethyl acetate extract containing indole compounds from (**B**): isolate Cbg70 and (**C**): isolate Zch127 after 4 days of incubation.

HPLC analysis was done to identify and quantify indole derivatives precisely. Several authentic indole derivatives (100 µM each, except IPA at 200 µM) were separated and their peaks were detected by HPLC using a C_18_ reverse column (Figure 6). Under the optimum conditions of analysis, the retention times of tryptophan (Trp), tryptamine (TAM), indole-3-acetamide (IAM), indole-3-lactic acid (ILA), indole-3-acetic acid (IAA), indole-3-acetonitrile (IAN), and indole-3-pyruvic acid (IPA) were 4.04, 4.25, 8.2, 11.9, 18, 24.8, and 36.4 min, respectively. Extracts from the selected rhizobacterial cultures of *Enterobacter* sp. isolates (Cbg70 and Zch127) in trypticase soy broth medium supplemented with 0.5 mM Trp were analyzed using HPLC and indole derivatives were identified and quantified after two and four days of incubation. Typically, 14–15 peaks, were identified and confirmed based on their retention time (Rt) and co-elution with authentic standards (Figure 6A). When comparing extract of the isolate Cbg70 to the authentic standards (Figure 6B), it showed peaks 4, 8, 10, 12, and 14 that co-eluted with Trp, IAM, ILA, IAA, and IPA, respectively, with variable concentrations. The bacterial culture of isolate Zch127 was analyzed by HPLC (Figure 6C). Peaks 4, 8, 10, 12, 13, and 14 co-eluted with Trp, IAM, ILA, IAA, IAN, and IPA, respectively. Other peaks remain to be determined. Indole acetic acid concentrations increased by increasing time of incubation and consumption of more tryptophan by the tested isolates (Table 3).

**Figure 7:**
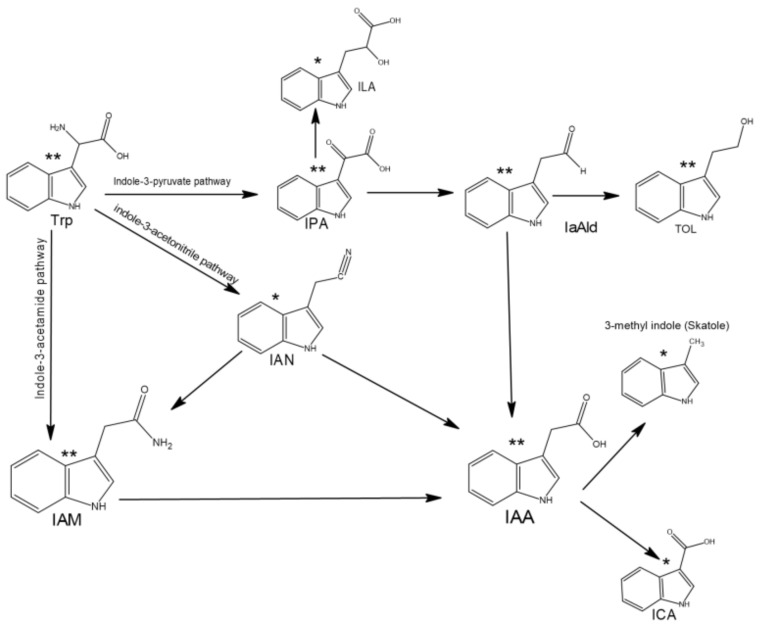
Proposed pathways of indole acetic acid (IAA) synthesis in the supernatant extracts of the *Enterobacter* sp. cultures, where ** means that the compound was detected in both cultures while * means that it was detected in the extract of Zch127 culture only, where Trp: tryptophan, IAM: indole-3-acetamide, IAN: indole-3-acetamide, IPA: indole-3-pyruvic acid, ILA: indole-3-lactic acid, IaAld: indole-3-acetaldehyde, TOL: indole-3-ethanol and ICA: indole-3-carboxylic acid.

Intermediates that belong to different pathways were detected in both extracts, including IAM, which is the key intermediate in the indole acetamide pathway, in addition to TOL, indole-3-acetaldehyde (IaAld), IPA, and ILA, which are the products in indole pyruvic acid pathway, whereas IAN (the product of indole acetonitrile pathway) was detected only in extract Zch127 (Figure 7). 

The presence of multiple pathways for tryptophan catabolism in the same microorganism was reported previously in *Rubrivivax benzoatilyticus* [29] and *Pseudomonas putida* [30]. Genome of *E. cloacae* subsp. cloacae NCTC 9394 and *E. cloacae* ECNIH5 had been shown to possess the *ipdC* gene (The key gene for indole pyruvate pathway) and the *iaaH* gene (The key gene of indole acetamide pathway), nitrilase enzyme which acts on indole-3-acetonitrile (The key gene of indole acetonitrile pathway) was previously reported in the genome of *Enterobacter cloacae* subsp. cloacae ATCC 13047 [31] which confirms the presence of multiple pathways for IAA biosynthesis in these bacteria. Indole 3-pyruvic acid pathway in *E. cloacae* was reported in previous studies [20,32,33]. Interestingly, the extract Zch127 showed higher antivirulence activity against tested isolates compared to Cbg70 which could be attributed to the presence of the three unique compounds IAN, methyl indole and I3CA. A previous study has revealed the high antibiofilm activity of IAN compared to other indole derivatives against pathogenic bacteria such as *Escherichia coli* O157:H7 and *P. aeruginosa* [9]. 2-methylindole (skatole), reduced biofilm formation of EHEC O157:H7 (ATCC 43894) by 52% [13]. Previous studies have reported the detection of enzymes involved in indole acetonitrile pathway in plant associated bacteria [34,35]. 

### 2.9. Effect of Synthetic IAA and IAN on P. mirabilis Virulence Traits

**Figure 8:**
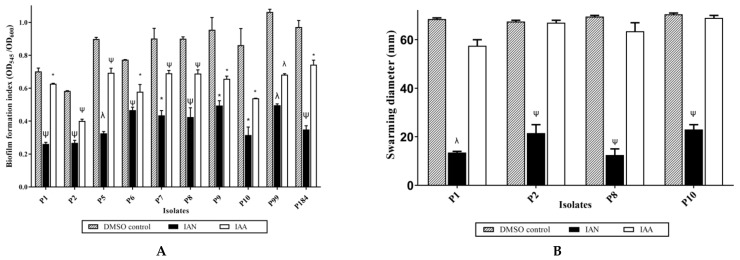
Effect of synthetic indole-3-acetonitrile (IAN) and indole-3-acetic acid (IAA) at concentration of 50 µg/mL on the virulence phenotype of *P. mirabilis* (**A**) Biofilm formation index of ten *P. mirabilis* isolates, (**B**) Swarming motility of four *P. mirabilis* isolates, Data represents the mean of at least 3 replicates, and error bars show standard error of the mean and statistical difference was evaluated by student’s *t*-test * *p* < 0.05; ^ψ^ *p* < 0.01; ^λ^ *p* < 0.001 compared with DMSO control.

The antivirulence activity of two of the detected compounds in the culture extract was confirmed by using synthetic standards against *P. mirabilis* isolates. The two compounds were IAA, detected in high concentration in the two extracts, and IAN, detected in Zch127 extract only. The antivirulence assays were performed using IAA and IAN in a concentration of 50 µg/mL which had no significant inhibitory effect on cell growth. Both IAA and IAN caused significant reduction in the biofilm formation by the 10 *P. mirabilis* isolates, but the highest antibiofilm effect was observed in samples grown in presence of IAN compared to those grown in IAA (Figure 8A).

Motility behavior was assessed by the swarming motility assay on four *P. mirabilis* isolates. IAN (detected in Zch127 extract only) showed inhibitory effect on the swarming motility of *P. mirabilis* isolates compared to the controls (Figure 8B) while IAA failed to inhibit the swarming motility.

## 3. Materials and Methods

### 3.1. Reagents and Standards 

All chemicals and solvents were purchased from Sigma–Aldrich (St. Louis, MO, USA), except L-tryptophan (Trp), indole-3-acetic acid (IAA), indole-3-lactic acid (ILA), and tryptamine (TAM), which were the products of Acros Organics (Morris Plains, NJ, USA), and indole-3-pyruvic acid (IPyA), which was purchased from Santa Cruz Biotechnology (Dallas, TX, USA). All chemicals and solvents used were analytical grade except those used in high-performance liquid chromatography (HPLC) and LC-MS which were of HPLC grade. Crude extracts stock (50 mg/mL) were prepared in 50% DMSO. Stock solutions of extracts and dimethyl sulfoxide (DMSO) control were subsequently diluted into media to yield the concentrations that are indicated in each experiment.

### 3.2. Clinical Strains and Growth Conditions

The antivirulence activity of crude indole extracts were assessed against ten *P. mirabilis* isolates recovered from urinary tract infections. Isolates were obtained from Kasr –El Ainy Hospital in a previous study [5] after the approval of the Ethics Committee in the Faculty of Pharmacy- October University for Modern Sciences and Arts (MSA) with ethics approval number (M1/EC1/2016PD). The study was also approved by the Ethics Committee in the Faculty of Pharmacy- Cairo University with ethics approval number MI (1739). The bacteria were routinely cultured aerobically at 37 °C in Luria–Bertani (LB) media. Susceptibility of these isolates to the respective antibiotics were carried out by disc diffusion method (Kirby-Bauer method) according to CLSI [28].

### 3.3. Isolation of Bacteria from Soil, Identification, and Screening for Indole Production

The rhizobacteria used in this study were isolated from the soil surrounding roots of plants in different locations in Egypt. Soil samples were transported in sterile polyethylene bags, secured between two ice packs to the laboratory [36].

One gram of soil sample was suspended in 20 mL of sterile saline and incubated at 30 °C and 200 rpm for 30 min. One milliliter of the soil suspension was then serially diluted (ten-fold) and cultured on nutrient agar followed by incubation at 30 °C for two days [37]. Collected isolates were screened for the production of indole derivatives using the quantitative method developed by Bric et al. [38] with modifications. Briefly, overnight cultures of the tested isolates, grown in Trypticase Soy Broth (TSB) medium, were adjusted to OD_600_ of 1 then diluted 1:100 in fresh TSB supplemented with L-tryptophan to a final concentration of 0.5 mM. Flasks were incubated at 30 °C with shaking (120 rpm) for 4 days. Quantitative estimation of indole derivatives in supernatants were carried out by the colorimetric assay using Salkowski’s reagent [39]. Isolates showing high production of indole derivatives were identified by the conventional microbiological methods followed by molecular analysis based on their 16s *rRNA* gene sequence. Target gene was amplified by the universal primers designed by Weisburg et al. [40] followed by sequencing using ABI 3730xl DNA Analyzer in Macrogen^®^ (Seoul, South Korea). Gene sequences were compared to sequences in the NCBI database using the Basic Local Alignment Search Tool (BLAST). Genetic diversity between selected isolates was analyzed using Molecular Evolutionary Genetics Analysis version 6.0 (MEGA 6). The phylogenetic tree was constructed by the maximum parsimony method [41]. 

### 3.4. Preparation of Crude Extracts from Enterobacter sp. Isolates

Indole producing isolates were grown under the same conditions used in the preliminary screening for indole production. Supernatant was acidified to pH 2.5 to 3.0 using HCl (1 N). Extraction of the metabolites from the acidified supernatant was carried out by shaking with double volumes of ethyl acetate (EtOAc) and that was repeated twice. The EtOAc fractions were pooled and dried under vacuum in a rotary flash evaporator (Heidolph, Germany) at 45 °C. A stock solution of 50 mg/mL was prepared by dissolving dried extract in 50% DMSO [42]. 

### 3.5. Determination of Minimum Inhibitory Concentration (MIC) of Crude Extract against P. mirabilis Isolates

Crude extracts from the supernatant of the selected isolates, were tested against the *P. mirabilis* isolates to determine their MIC according to the Clinical and Laboratory Standards Institute [28] guidelines. Bacterial cultures were added at initial inoculum density of 5×10^5^ CFU mL^−1^. Crude extracts were added with concentrations range 0.01–5 mg/mL. 

The effect of the sub-MICs of crude extract on the growth of *P. mirabilis* was determined by turbidimetric assay. Briefly, overnight cultures of *P. mirabilis* clinical strains in LB were diluted to achieve turbidity equivalent to OD_600_ of 1 and then diluted 1:100 in LB broth supplemented with the extract in a final concentration of half the MIC. The culture was then incubated at 37 °C with constant shaking at 250 rpm. Culture supplemented with DMSO at the same final concentration as the test was used as a control. The optical density of the respective bacterial growth at 600 nm was determined at different time intervals that ranged from 0 to 24 h [6].

### 3.6. Assessment of the Antivirulence Potential of Crude Extract against P. mirabilis

#### 3.6.1. Effect on Biofilm Formation 

The biofilm forming ability of the *P. mirabilis* isolates was determined by the crystal violet assay [43]. After incubation, microbial growth was determined by measuring the turbidity at 600 nm (OD_growth_) and the biofilm biomass was measured colorimetrically at 545 nm (OD_CV_). The biofilm formation was evaluated using a biofilm formation index [BFI]: (OD_CV_ Biofilm − OD_CV_ Control)/OD_growth_ [27]. Isolates were classified into non-adherent, weak, moderate and strong biofilm forming according to the semiquantitative classification of biofilm production as described by Naves et al. [44]. Effect of the extract on biofilm formation by *P. mirabilis* strains was estimated according to Labrecque et al. [45] with slight modifications. Briefly, overnight cultures of *P. mirabilis* strains were diluted to reach a cell density of 1.5 × 10^8^ CFU/mL and then diluted to 1:50 in LB broth. In each well, 100 µL of diluted cultures were added to equal volume of the extract at 2X of the required sub-MIC concentration in 96-well flat bottom polystyrene microtiter plate (Greiner Bio-one^®^, Germany). Culture with DMSO and blank medium served as positive and negative controls, respectively. Plates were incubated at 37 °C for 24 h without agitation. Biofilm was stained by crystal violet (1%). Unbound stain was removed and the wells were washed with 200 µL of sterile distilled water. The water was removed and the plates were air dried. Finally, stain was solubilized in glacial acetic acid (33%).

#### 3.6.2. Effect on the Swarming and Swimming Behavior 

One milliliter of an overnight culture of *P. mirabilis* in LB broth was centrifuged and the resulting pellet was washed twice by 1 mL of saline (0.9%), and finally suspended in saline to reach a concentration equivalent to OD_600_ of 1. To test the effect of the extract on the swarming motility, five microliters of the adjusted suspension was pipetted onto the dry surface of LB plates (2% agar) containing sub-MIC of the extract. After 16 h of incubation at 37 °C, the diameter of the swarm circle was measured in mm and compared to motility on control plate containing equivalent concentration of DMSO to test plates [46]. 

For swimming assay, the adjusted overnight *P. mirabilis* cultures were stabbed into the center of a dried LB swimming agar plate (0.4% w/v agar) with sub-MIC concentrations. After overnight incubation at 37 °C, the swimming zones diameters were measured in mm and the results were compared to control plates [47].

#### 3.6.3. Effect on Urease Production

The effect of the extracts on the urease production by *P. mirabilis* was carried out using liquid Christensen’s medium, with added urea at concentration of 20 g/L. Inoculum used was prepared as described previously (for swarming and swimming test). One hundred µL of microbial suspension were inoculated in 10 mL of the medium containing the extracts at sub-MIC and incubated for 3 h at 37 °C. After incubation, bacterial cells were removed by centrifugation and the change in color of supernatant was measured at wavelength 570 nm and compared to the color of the control without extract [48]. 

#### 3.6.4. Effect on the Minimum Inhibitory Concentration (MIC) of Antibiotics 

The effect of crude extract on bacterial sensitivity to antibiotics was determined by measuring the change in the MIC of antibiotics upon combination with extract. Antibiotics that belong to different classes were selected including ceftriaxone, amikacin and ciprofloxacin. MICs were determined by microbroth dilution method according to the Clinical and Laboratory Standards Institute guidelines [28]. MIC values were recorded in absence and presence of sub-MICs of crude extract. DMSO, equivalent to those present in crude extract, was added in the extract free wells. [6]. The synergistic and antagonistic activity of this combination was reflected by decrease and increase in MIC values, respectively, compared to untreated isolates.

### 3.7. HPLC and LC-MS/MS Analysis of the Microbial Extract

Indole compounds in the EtOAc extract were analyzed using Waters^®^ 600E HPLC (Milford, MA, USA) equipped with a Nucleosil 120-5 C_18_ reverse column (5 µm, 250U4 mm) from Richard Scientific (Novato, CA, USA). Detection wavelength was 280 nm, and confirmed by retention time and co-migration (spiking with an authentic standard). Further verification and quantification of indole derivatives were performed using authentic standards of Trp, indole-3-acetic acid (IAA), indole-3-ethanol (TOL), indole-3-pyruvic acid (IPA), indole-3-acetamide (IAM), indole-3-acetonitrile (IAN), tryptamine (TAM), and indole-lactic acid (ILA). The authentic indole compounds were used in concentration of 100 µM each, except IPA which were used at 200 µM [49]. The concentration was estimated by the determination of the area under the curve (AUC).

$$\text{Concentration}\;\text{of}\;\text{the}\;\text{unknown}=\frac{(\text{AUC}\;\text{of}\;\text{the}\;\text{unknown}\;\text{sample}-\text{AUC}\;\text{of}\;\text{the}\;\text{standard})\times \text{Conc}.\;\text{of}\;\text{the}\;\text{standard}}{\text{AUC}\;\text{of}\;\text{the}\;\text{standard}}$$

The *Enterobacter* sp. extracts were analyzed using liquid chromatography-mass spectrometry (LC-MS). Electrospray Ionization Mass Spectrometry (ESI-MS) positive and negative ion acquisition mode were carried out on a Waters^®^ Xevo TQD (Milford, MA, USA), coupled to mass spectrometer Column: (ACQUITY UPLC-BEH C_18_ 1.7 µm − 2.1 × 50 mm column) with flow rate of 0.2 mL/ min. Solvent system: consisted of gradient mobile phase (A): Water containing 0.1% formic acid, (B): Methanol containing 0.1% formic acid. The sample (100 μg/mL) solution was dissolved in methanol, and filtered then subjected to LC-MS analysis [29].

The parameters for analysis were carried out using positive and negative ion mode as follows: source temperature 150 °C, cone voltage 30 eV, capillary voltage 3 kV, desolvation temperature 440 °C, cone gas flow 50 L/h, and desolvation gas flow 900 L/h. Full scan MS was between *m/z* 100–1000. The peaks and spectra were processed using the Masslynx 4.1 software and tentatively identified by comparing its retention time (Rt) and mass spectrum, and mass fragmentation pattern reported in Mass Bank (http://www.massbank.jp), and PubChem (https://pubchem.ncbi.nlm.nih.gov/) (accessed on 15 August 2020).

### 3.8. Statistical Analysis

All experiments were repeated at least three times. Statistical analysis of the data obtained from the studies was conducted with GraphPad Prism version 8.0.0 for Windows, (San Diego, CA, USA) software. Statistical tests used were Student’s *t*-test, and two-way ANOVA (analysis of variance) where appropriate. *p* ≤ 0.05 was considered as significant. 

## 4. Conclusions

The current work has revealed that indole derivatives extracted from the rhizobacteria *Enterobacter* sp. have promising antivirulence and antibiofilm activity against *P. mirabilis* which is one of the important causative agents of complicated urinary tract infections. The results of the HPLC and LC-MS analysis of the crude extract suggest that there is more than one metabolic pathway for production of indole acetic acid in *Enterobacter* sp. The metabolic pattern of *Enterobacter* sp. differs according to the soil environment from which it was isolated. The IAN detected in the Zch127 could be responsible for the significant anti-swarming and anti-urease activity of this extract compared to Cbg70 extract.

## Figures and Tables

**Table 1:** Fold changes in minimum inhibitory concentration of selected antibiotics in presence of indole derivatives crude extracts at sub-MIC against *Proteus mirabilis* isolates.

Antibiotic	Isolate No.	MIC	MIC with Extract Cbg70 ^a^	Fold Change in MIC with Cbg70	MIC with Extract Zch127 ^b^	Fold Change in MIC with Zch127
**Ceftriaxone**	**P1**	64(R)	32(R)	−2X	16(R)	−4X
	**P2**	16(R)	16(R)	−	4(R)	−4X
	**P5**	16(R)	16(R)	−	8(R)	−2X
	**P6**	32(R)	32(R)	−	32(R)	−
	**P7**	4(R)	8(R)	2X	4(R)	−
	**P8**	128(R)	128(R)	−	64(R)	−2X
	**P9**	64(R)	64(R)	−	64(R)	−
	**P10**	8(R)	8(R)	−	8(R)	−
	**P99**	2(I)	1(S)	−2X	1(S)	−2X
	**P184**	16(R)	16(R)	−	8(R)	−2X
						
**Ciprofloxacin**	**P1**	16(R)	16(R)	−	16(R)	−
	**P2**	16(R)	32(R)	2X	64(R)	4X
	**P5**	32(R)	64(R)	2X	64(R)	2X
	**P6**	32(R)	64(R)	2X	32(R)	−
	**P7**	4(R)	8(R)	2X	8(R)	2X
	**P8**	64(R)	64(R)	−	64(R)	−
	**P9**	64(R)	64(R)	−	64(R)	−
	**P10**	16(R)	8(R)	−2X	32 (R)	2X
	**P99**	8(R)	8(R)	−	8(R)	−
	**P184**	64(R)	32(R)	−2X	64(R)	−
						
**Amikacin**	**P1**	4(S)	4(S)	−	0.5(S)	−8X
	**P2**	8(S)	8(S)	−	2(S)	−4X
	**P5**	8(S)	16(S)	2X	1(S)	−8X
	**P6**	8(S)	16(S)	2X	2(S)	−4X
	**P7**	4(S)	8(S)	2X	0.5(S)	−8X
	**P8**	8(S)	32(I)	4X	0.5(S)	−16X
	**P9**	16(S)	16(S)	−	0.5(S)	−32X
	**P10**	4(S)	4(S)	−	4(S)	−
	**P99**	4(S)	16(S)	4X	1(S)	−4X
	**P184**	8(S)	32(S)	4X	0.5(S)	−16X

^a^ sub-MIC = 0.08 mg/mL, ^b^ sub-MIC = 0.6 mg/mL. (−) before fold of change denotes decrease in MIC. Results of MIC was interpreted according to CLSI [28] into Resistant (R), Intermediate (I), Sensitive (S).

**Table 2:** LC-MS identified peaks of the extract from isolates Cbg70 and Zch127and their fragmentation pattern.

**Cbg70**			
* Rt (min)	**Peak No.**	**Mass (m/z) Fragmentation**	**Indole Derivative**
2.87	13	205[M+H] ^+^ (205,159,144,143,130,117,115)	Tryptophan
8.01	30	176[M+H] ^+^ (176,130, 103)	Indole-3-acetic acid (IAA)
8.01	33	162[M+H] ^+^ (162,144,143,117,115)	Indole-3- ethanol (TOL)
8.3	33	146[M+H] ^+^ (146,118,117,91)	Indole-3-aldehyde
11.2	45	205[M+2H] ^+^ (205,142, 139,117)	Indole-3-pyruvic acid
11.88	48	198[M+K] ^+^ (198,133,118)	Indole-3-acetaldehyde (IAld)
1.07	3	188[M-H]^−^ (188,128, 59)	Indole-3-propionic acid
4.62	17	158[M-H]^−^ (158,130, 117)	Indole-3- acetaldehyde
6.76	28	174[M-H]^−^ (174,130, 128)	Indole-3-acetic acid (IAA)
7.43	31	160[M-H]^−^ (160,130)	Indole-3-ethanol (TOL)
7.77	31	173[M-H]^−^ (174,131, 130, 111)	Indole-3-acetamide
**Zch127**			
**Rt (min)**	**Peak No.**	**Mass (m/z) Fragmentation**	**Indole Derivative**
1.31	10	132[M+H] ^+^ (132,86, 69)	3-methyl indole (skatole)
1.98	7	162[M+H] ^+^ (162,144,120)	Indole-3-carboxylic acid (I3CA)
6.66	19	176[M+H] ^+^ (176,130, 118, 103, 77)	Indole-3-acetic acid (IAA)
6.93	19	245[M+K] ^+^ (245,174, 173, 130)	Indole-3-lactic acid (ILA)
8.01	26	162[M+H] ^+^ (162,144,143,117,115)	Indole-3-ethanol (TOL)
9.04	37	205[M+2H] ^+^ (205,158,130,87,72)	Indole-3-pyruvic acid
13.1	76	199[M+H+K] ^+^ (199,133,117)	Indole-3- acetaldehyde
14.46	86	196[M+H+K] ^+^ (196,131,117, 79)	Indole-3-acetonitrile
2.84	9	203[M-H]^−^ (203, 158, 146, 142, 132,118, 116)	Tryptophan
6.27	18	173[M-H]^−^ (173, 143, 130)	Indole-3-acetamide (IAM)
8.72	50	202[M-H]- (202, 158, 130)	Indole-3-pyruvic acid
16.21	97	130[M-H]- (130, 115.4)	3-methyl indole (skatole)

* Rt: Retention time.

**Table 3:** The concentration (µM) of the identified compounds, in extracts Cbg70 and Zch 127 after 2 and 4 days of incubation, using HPLC confirmed by co-elution with the authentic indole derivative.

Compound	Rt	Cbg70 2 days (µM)	Cbg70 4 days (µM)	Zch127 2 days (µM)	Zch127 4 days (µM)
Tryptophan	4.040	44.226	36.648	40.798	9.866
IAM	8.221	22.800	25.120	19.120	22.880
ILA	11.984	21.465	24.148	24.045	10.939
IAA	18.005	99.915	199.680	117.241	321.278
IAN	24.830	0	0	4.407	19.729
IPA	36.380	19.428	24.390	24.978	7.232

## Data Availability

Data is contained within the article or supplementary material.

## Supplementary Materials

The following are available online at https://www.mdpi.com/article/10.3390/antibiotics10040363/s1, Table S1: Concentration of IAA produced by the bacterial isolates after 4 days of incubation in trypticase soy broth supplemented with 100 µg/mL (0.5mM) tryptophan, Figure S1: Evolutionary relationships between *Enterobacter* sp. Cbg70 and Zch127 with related strains, Figure S2: Effect of culture extract of Cbg70 on the growth of 10 *P. mirabilis* isolates, Figure S3: Effect of culture extract of Zch127 on the growth of 10 *P. mirabilis* isolates, Figure S4 and S5: The effect of extract Zch127 on *P. mirabilis* isolates swarming motility, Figure S6: The effect of extract Zch127 on *P. mirabilis* isolates swimming motility, Figure S7: LC-MS chromatograms from ESI positive and negative modes of the extracted compounds, Figure S8: Liquid chromatography–mass spectrometry (LC-MS) fragmentation analysis of targeted indole derivatives in Cbg70 at positive mode, Figure S9: Liquid chromatography–mass spectrometry (LC-MS) fragmentation analysis of targeted indole derivatives in Cbg70 at negative mode, Figure S10: Liquid chromatography–mass spectrometry (LC-MS) fragmentation analysis of targeted indole derivatives in Zch127 at positive mode, Figure S11: Liquid chromatography–mass spectrometry (LC-MS) fragmentation analysis of the targeted indole derivatives in Zch127 at negative mode.
