# Supplementary material for: Indole Derivatives Obtained from Egyptian Enterobacter sp. Soil Isolates Exhibit Antivirulence Activities against Uropathogenic Proteus mirabilis
Source: Antibiotics (Basel). 2021 Mar 29;10(4):363. doi: 10.3390/antibiotics10040363 (PMC8065651; doi:10.3390/antibiotics10040363)
Supplement: Supplementary file 1 [file antibiotics-10-00363-s001.pdf]

Table S1. Concentration of IAA produced by the bacterial isolates after 4 days of incubation in trypticase soy broth supplemented with 100 µg/mL (0.5mM) tryptophan.

Isolate no.	Soil source	Gram Reaction	Indole Absorbance mean ± SD (OD 530nm)	Indole production mean±SD (µg/mL)	Indole production mean±SD (µM)
**G1r17**	Garden 1	Positive	0.789±0.07	72.85±7.0137	416.28±40.07
**G2r21**	Garden 2	Positive	0.373± 0.009	33.613± 0.867	192.07±4.954
**G4r26**	Garden 4	Positive	0.655± 0.07	60.169± 7.338	343.822±41.931
**Mz55**	Maize	Positive	0.69± 0.002	63.518± 0.2001	363.96±1.143
**Cbg70**	Cabbage	Negative	0.56±0.03	51.348±2.868	293.417±16.38
**Cbg73**	Cabbage	Positive	0.53±0.04	48.446±4.13	276.834±23.6
**G2p78**	Green peas 2	Positive	0.809±0.086	74.697±8.138	426.84±46.5
**G1p98**	Green peas 1	Positive	0.79±0.07	72.404±5.962	413.73±34.06
**Pt101**	Potato	Negative	0.524±0.05	47.806±5.46	273.177±31.2
**Tf110**	Egyptian clover	Negative	0.634±0.03	58.185±3.47	332.485±19.82
**Tf112**	Egyptian clover	Negative	0.712± 0.08	65.59± 7.67	374.8±43.828
**Zch127**	Zucchini	Negative	0.581± 0.044	53.235± 4.202	304.2±24.011
**Pt128**	Potato	Negative	0.498±0.05	45.358±3.736	259.188±21.344

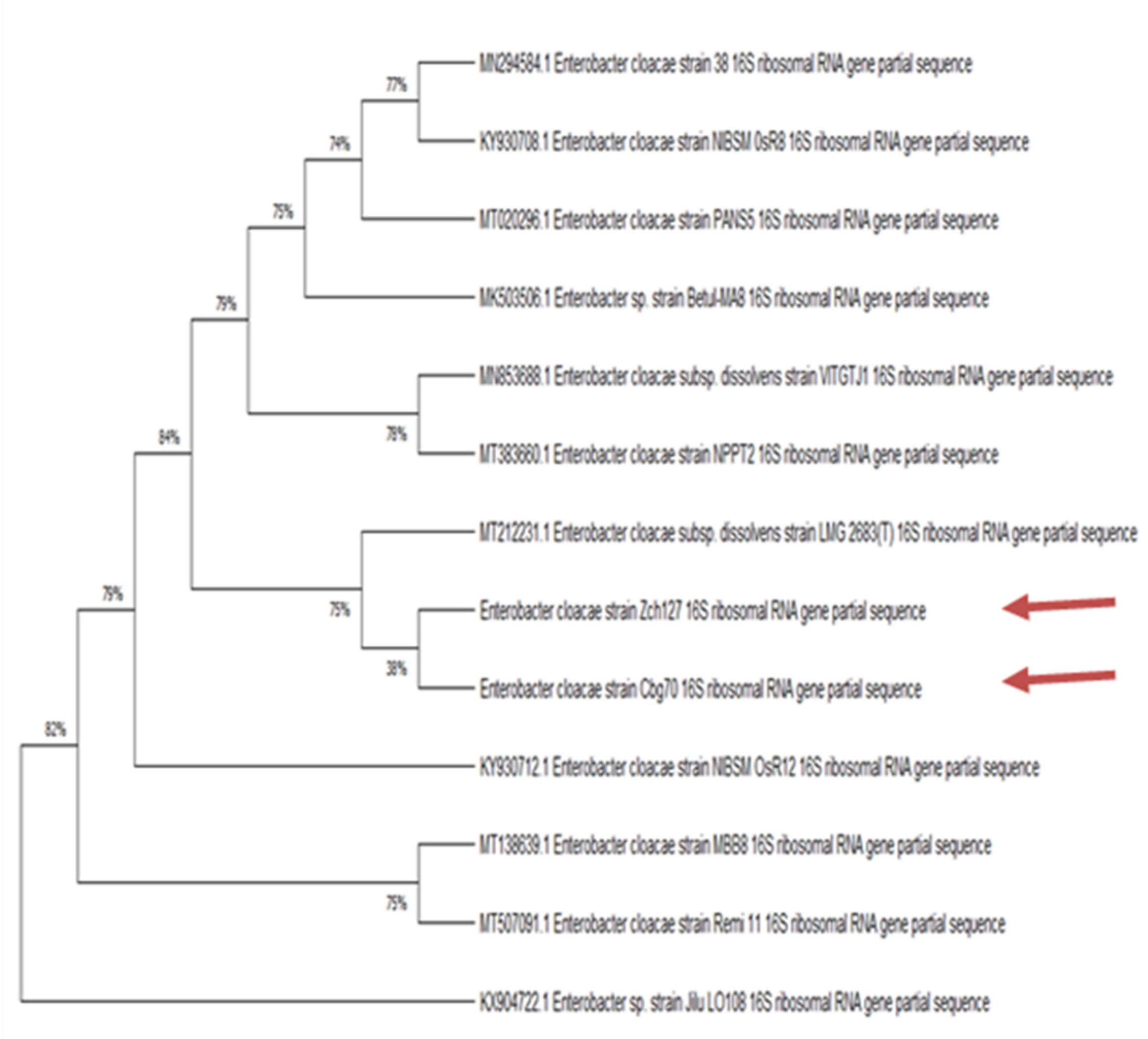

Figure S1. Evolutionary relationships between *Enterobacter cloacae* Cbg70 and Zch127 with related strains. Phylogenetic tree of isolates Cbg70 and Zch127 among neighborhood strains using Molecular Evolutionary Genetics Analysis version 6.0 (MEGA 6). The phylogenetic tree was constructed by maximum parsimony method.

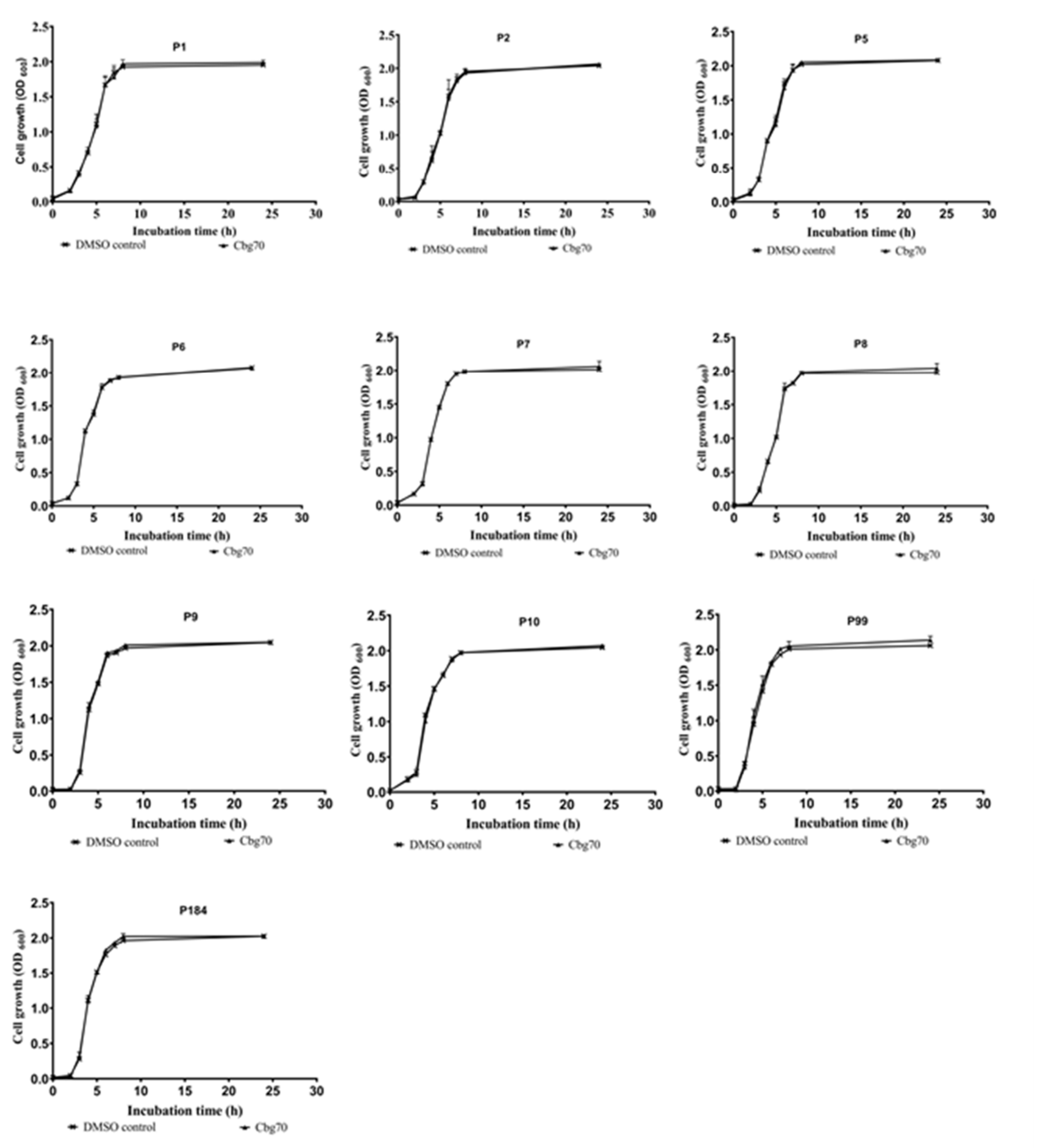

Figure S2. Effect of culture extract of Cbg70 on the growth of 10 *P. mirabilis* isolates. Growth was measured as OD in presence of sub-MIC concentration of 0.5X MIC extract Cbg70 (0.08 mg/mL), cells were grown in LB media at 37°C and 250 rpm. Bacterial culture supplemented with DMSO in the same concentration as extract served as control. Each experiment was performed using three different cultures, and average data set is shown.

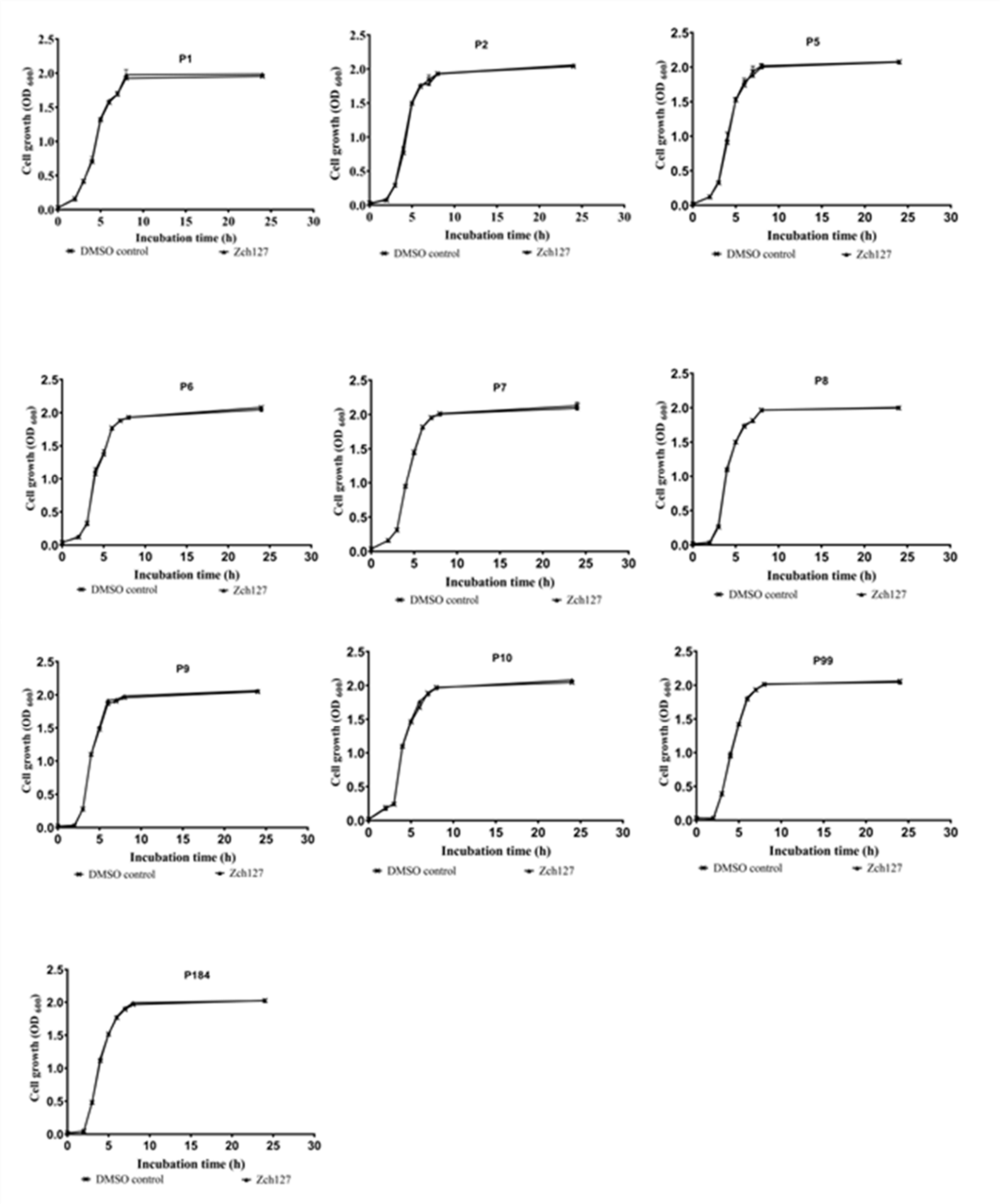

Figure S3. Effect of culture extract of Zch127 on the growth of 10 *P. mirabilis* isolates. Growth was measured as OD in presence of sub-MIC concentration of 0.5X MIC extract Zch127 (0.6 mg/mL), cells were grown in LB media at 37°C and 250 rpm. Bacterial culture supplemented with DMSO in the same concentration as extract served as control. Each experiment was performed using three different cultures, and average data set is shown.

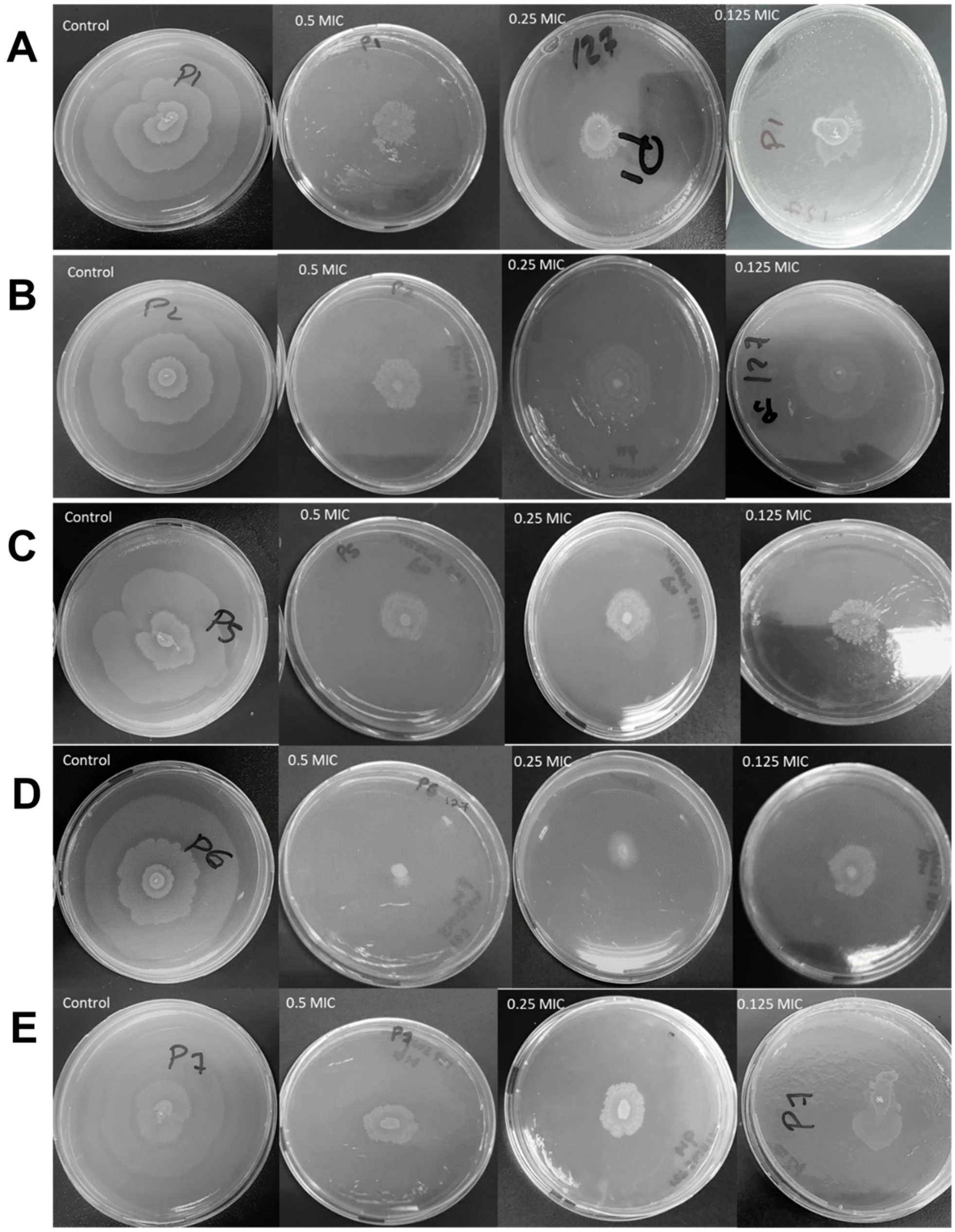

Figure S4. The effect of extract Zch127 on *P. mirabilis* isolates swarming motility, (A):P1, (B):P2, (C):P5, (D):P6, (E):P7, the left plate is the control *P. mirabilis* isolate and the right plate is treated with sub-MIC concentrations (0.6, 0.3 and 0.15 mg/mL) (0.5X, 0.25X and 0.125X MIC) of extract Zch127.

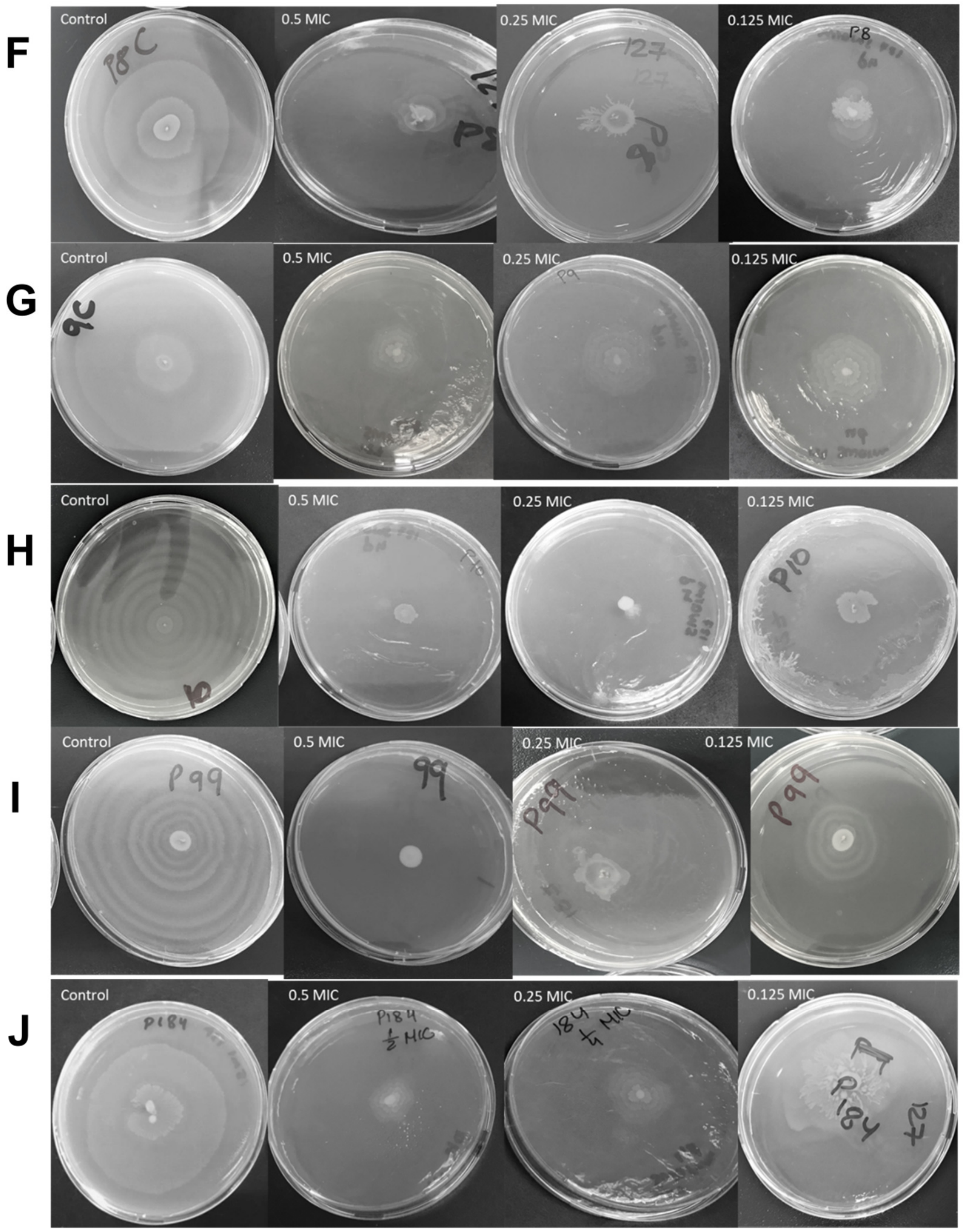

Figure S5. The effect of extract Zch127 on *P. mirabilis* isolates swarming motility, (F):P8, (G):P9, (H):P10, (I): P99, (J):P184, the left plate is the control *P. mirabilis* isolate and the right plate is treated with sub-MIC concentrations (0.6, 0.3 and 0.15 mg/mL) (0.5X, 0.25X and 0.125X MIC) of extract Zch127.

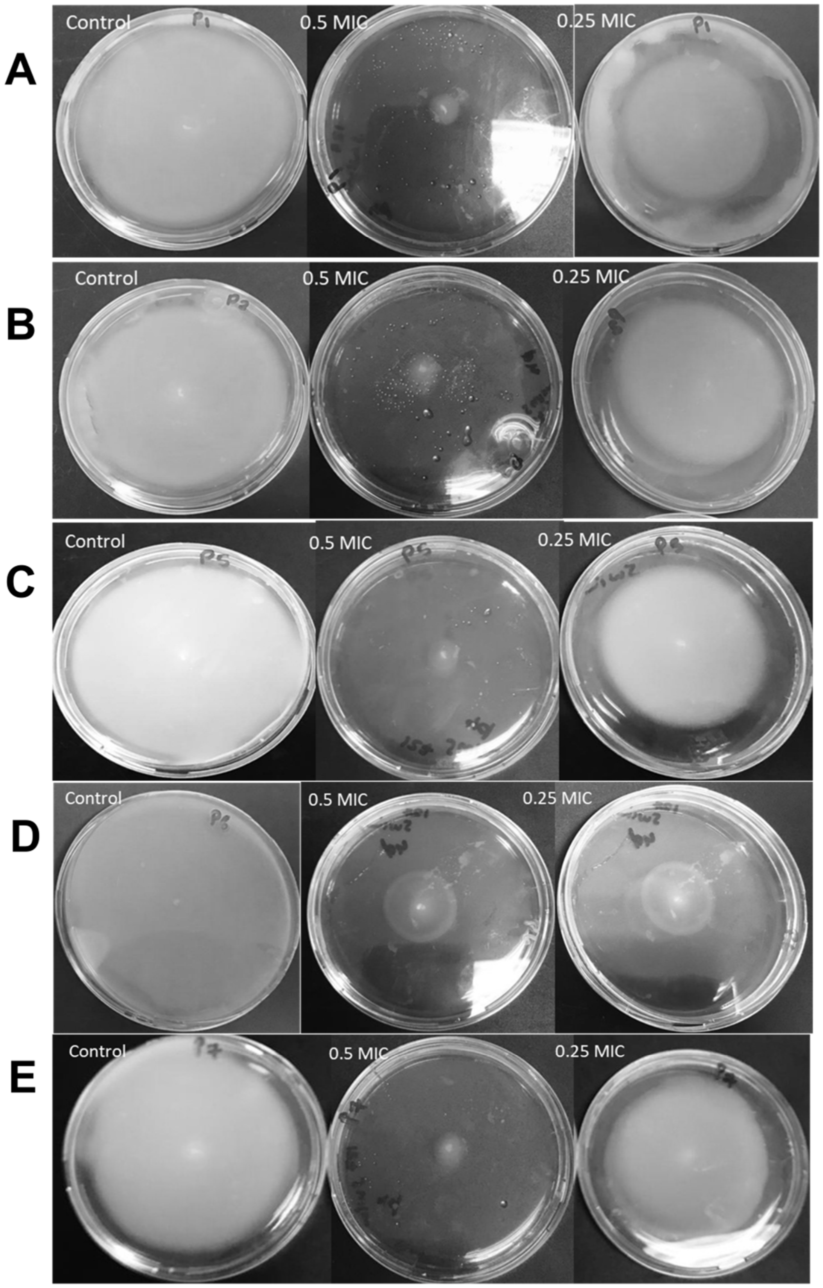

Figure S6. The effect of extract Zch127 on *P. mirabilis* isolates swimming motility, (A):P1, (B):P2, (C):P5, (D):P6, (E):P7, the left plate is control *P. mirabilis* isolate and the right plate is treated with sub-MIC concentrations (0.6 and 0.3 mg/mL) (0.5X and 0.25X MIC) of extract Zch127.

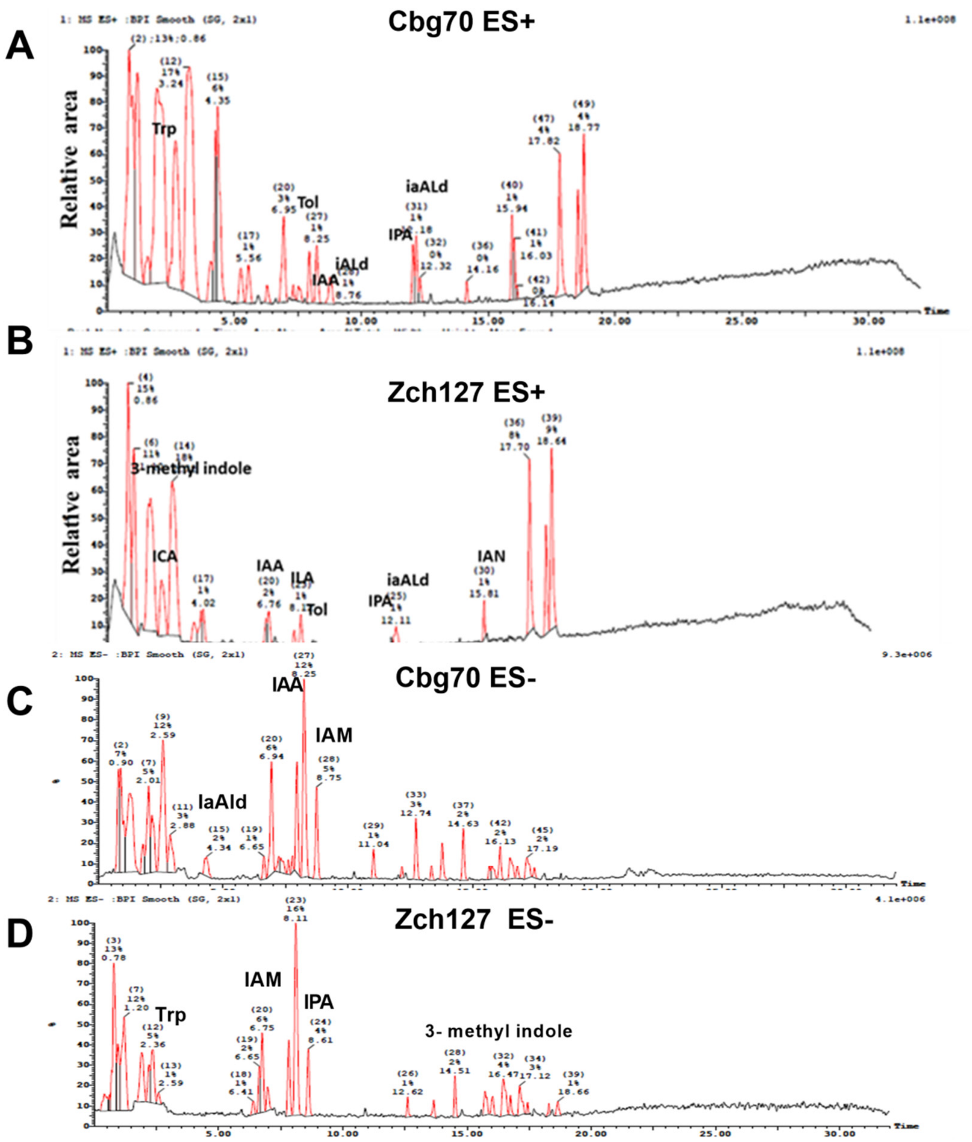

Figure S7. LC-MS chromatograms from ESI positive and negative modes of the extracted compounds. The chromatogram represents the extracted cultures from (A) isolate Cbg70 and (B) isolate Zch127 at positive mode and (C) isolate Cbg70 and (D) isolate Zch127 at negative mode after 4 days of incubation where Trp: tryptophan, Tol: indole-3-ethanol, IAA: indole-3-acetic acid, IALd: indole aldehyde, IPA: indole-3-pyruvic acid, iaALd: indole-3-acetaldehyde, ICA: indole-3-carboxylic acid, ILA: indole-3-lactic acid and IAN: indole-3-acetonitrile.

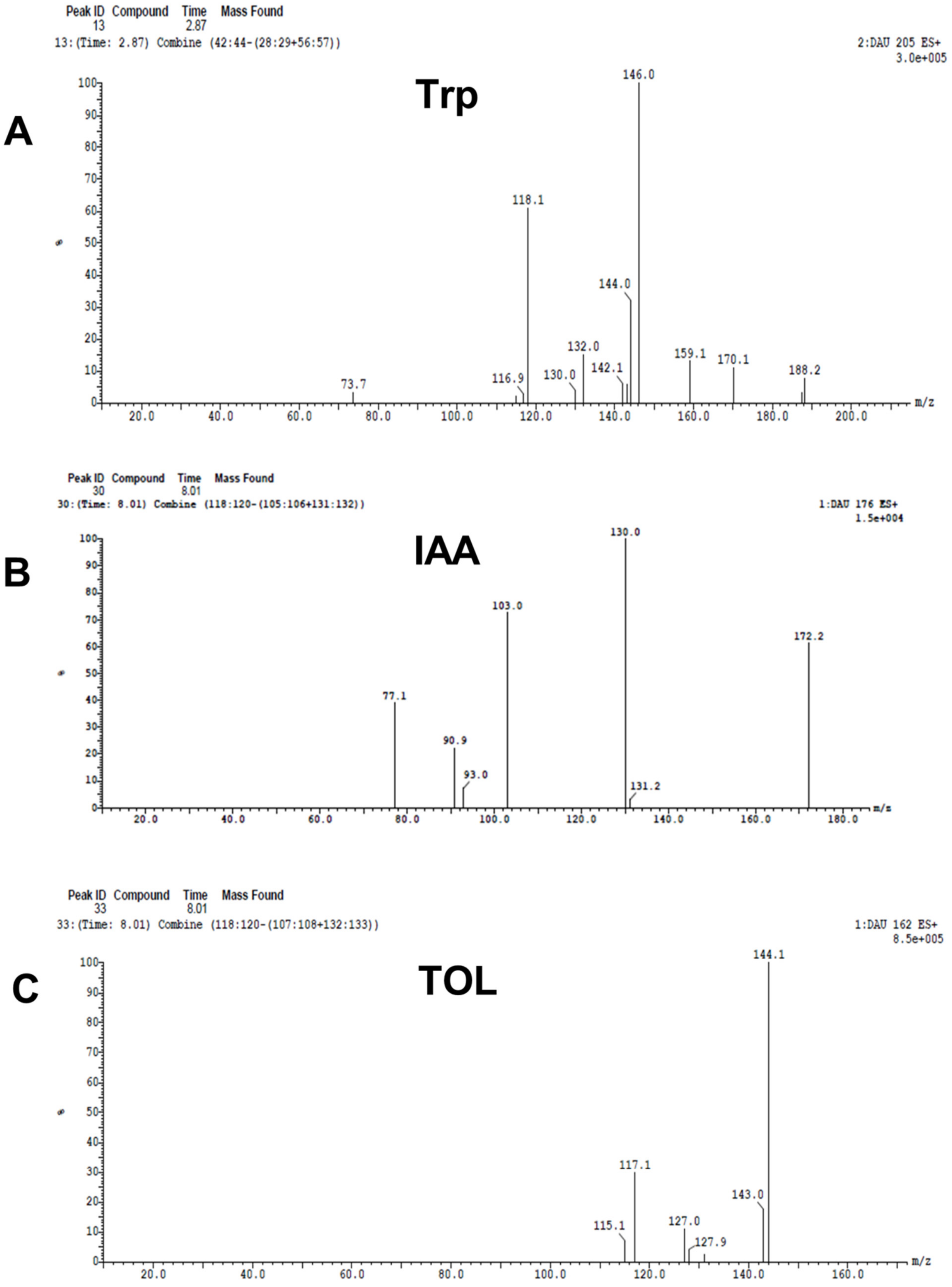
A
Trp
Peak ID Compound Time Mass Found
13 2.87
13:(Time: 2.87) Combine (42:44-(28:29+56:57))
2:DAU 205 ES+
3.0e+005
%
146.0
118.1
144.0
132.0
159.1
170.1
188.2
116.9
130.0
142.1
73.7
m/z
B
IAA
Peak ID Compound Time Mass Found
30 8.01
30:(Time: 8.01) Combine (118:120-(105:106+131:132))
1:DAU 176 ES+
1.5e+004
%
130.0
103.0
172.2
77.1
90.9
93.0
131.2
m/z
C
TOL
Peak ID Compound Time Mass Found
33 8.01
33:(Time: 8.01) Combine (118:120-(107:108+132:133))
1:DAU 162 ES+
8.5e+005
%
144.1
117.1
143.0
127.0
115.1
127.9
m/z

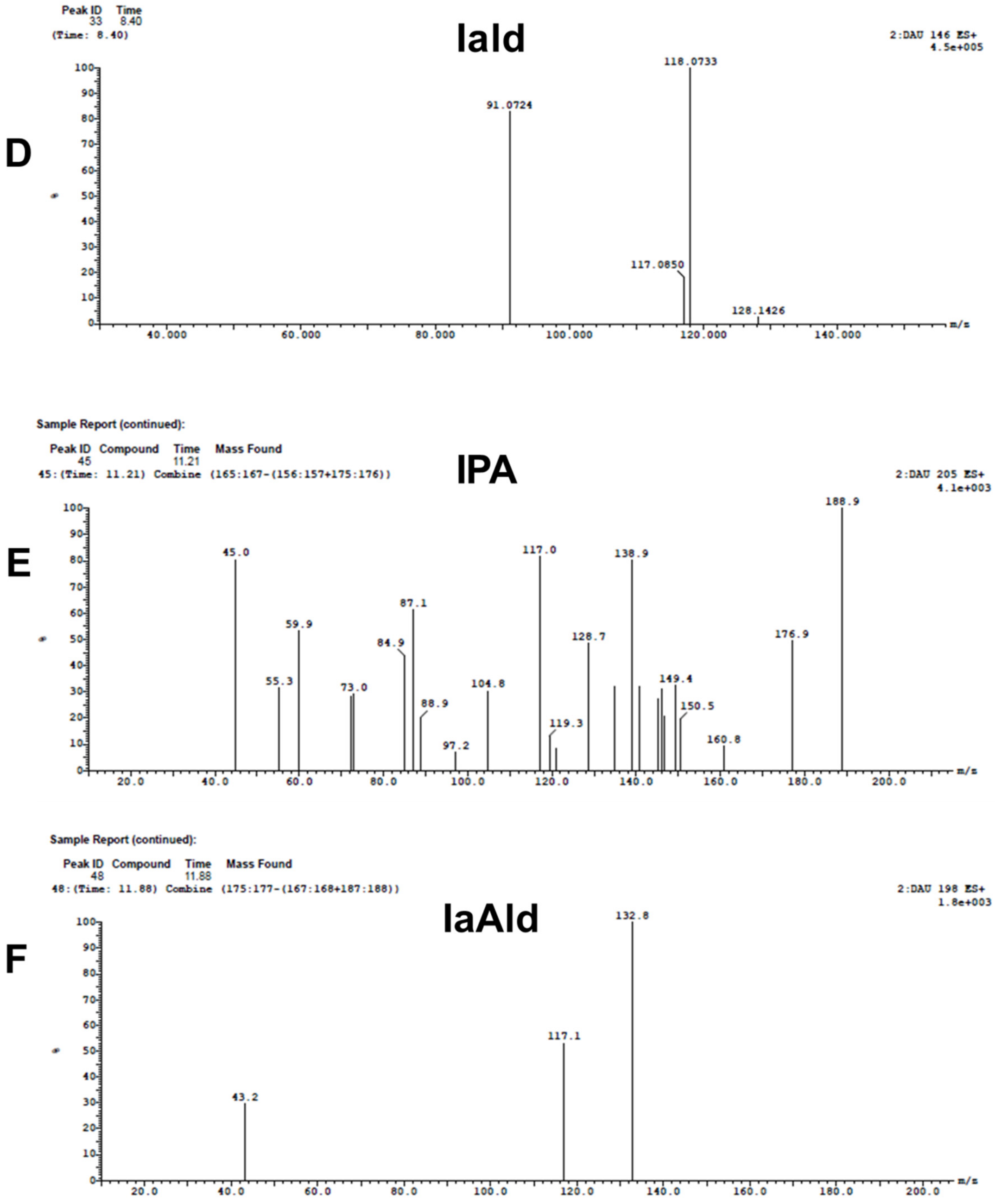

Figure S8. Liquid chromatography–mass spectrometry (LC-MS) fragmentation analysis of targeted indole derivatives in Cbg70 at positive mode; (A) Trp: tryptophan, (B) IAA: indole-3-acetic acid, (C) TOL: indole-3-ethanol, (D) indole-3-aldehyde (E) IPA: indole-3-pyruvic acid and (F) IAld: indole-3-acetaldeyde.

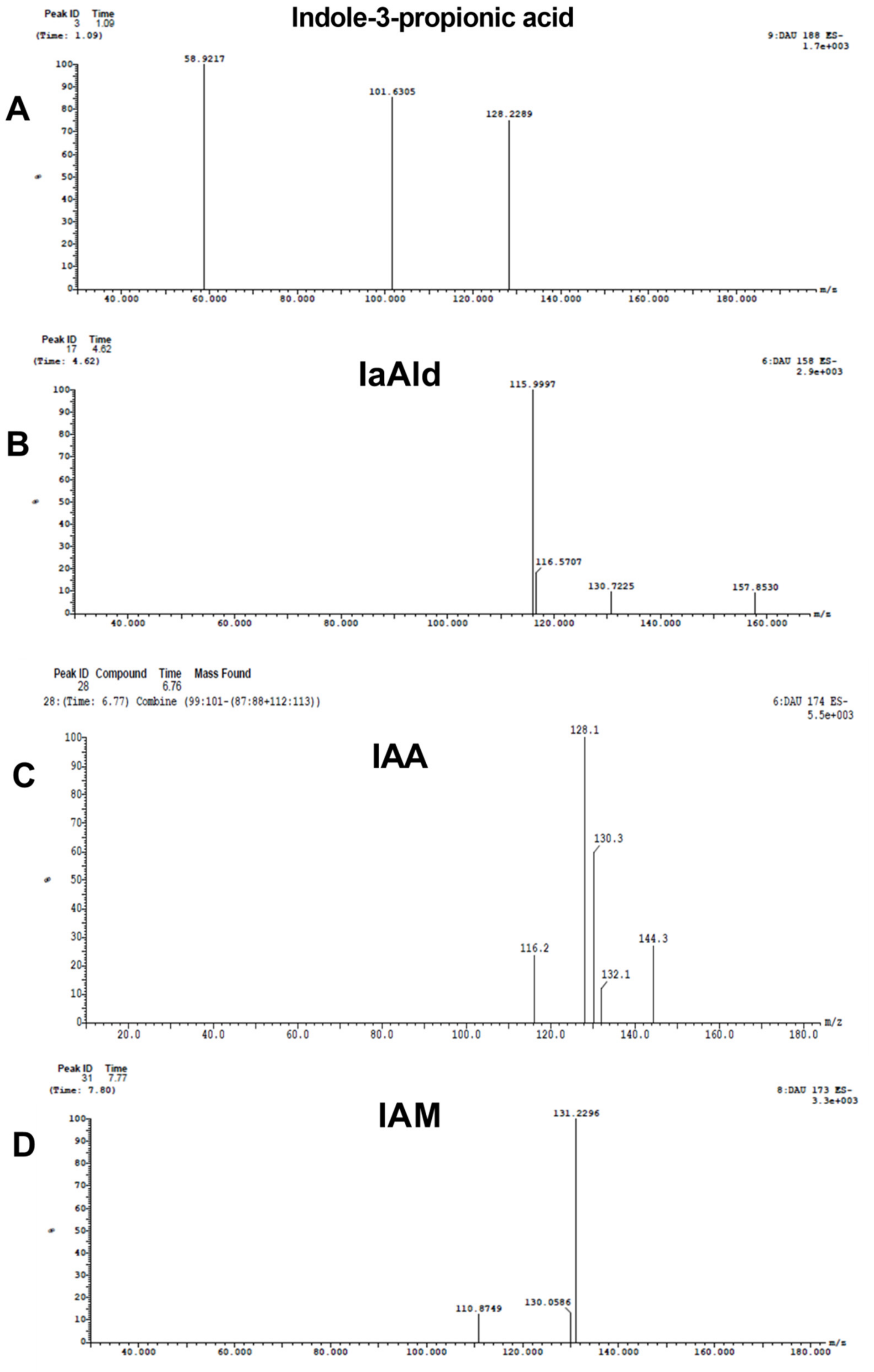

Figure S9. Liquid chromatography–mass spectrometry (LC-MS) fragmentation analysis of targeted indole derivatives in Cbg70 at negative mode; (A) Indole-3-propionic acid, (B) IAld: indole acetaldehyde, (C) IAA: indole-3-acetic acid and (D) IAM: indole-3-acetamide.

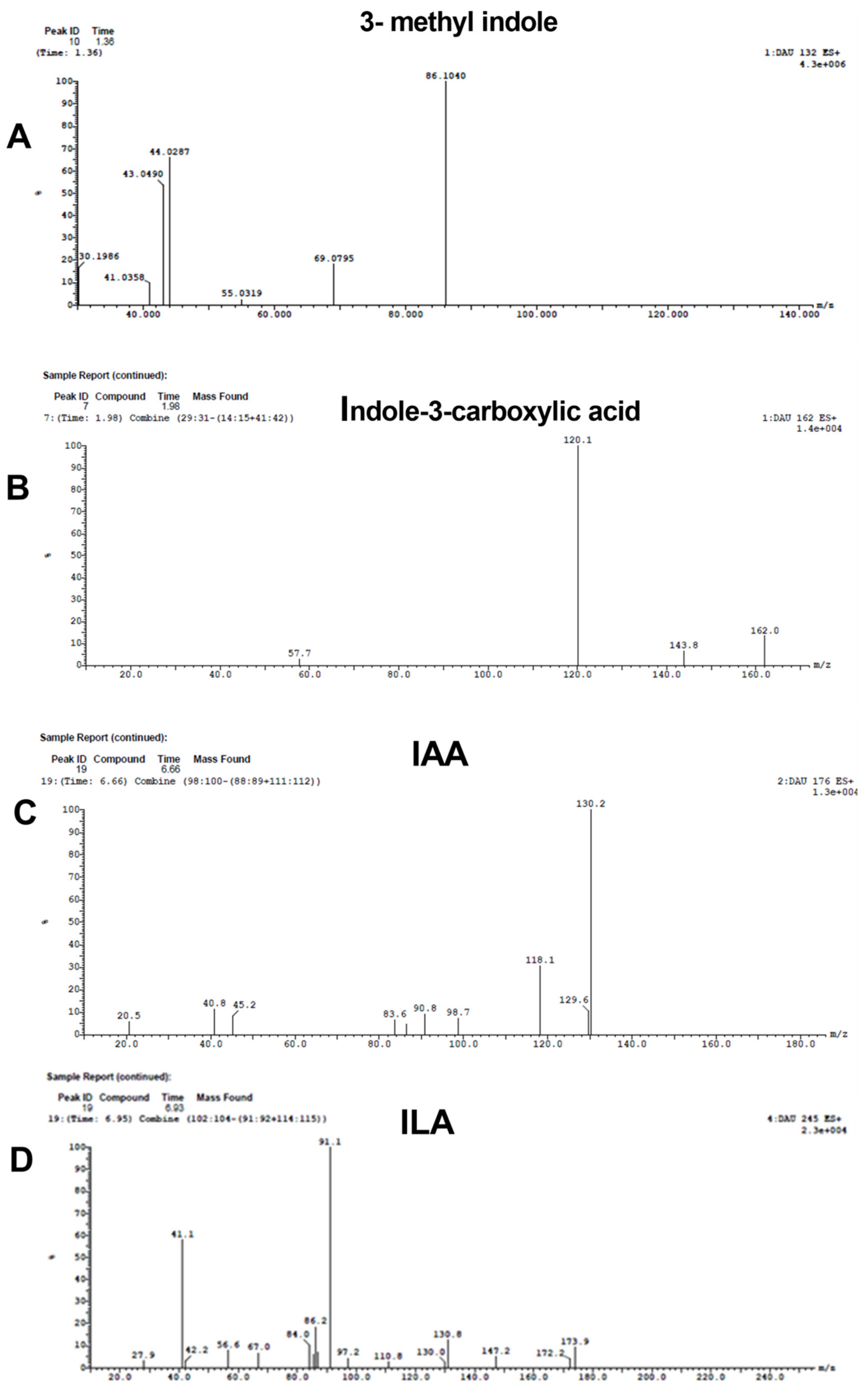
3- methyl indole
A
Peak ID Time
10 1.36
(Time: 1.36)
1:DAU 132 ES+
4.3e+006
86.1040
44.0287
43.0490
30.1986
41.0358
55.0319
69.0795
m/z
40.000
60.000
80.000
100.000
120.000
140.000
Sample Report (continued):
Peak ID Compound Time Mass Found
7 1.98
7:(Time: 1.98) Combine (29:31-(14:15+41:42))
Indole-3-carboxylic acid
B
1:DAU 162 ES+
1.4e+004
120.1
143.8
162.0
57.7
m/z
20.0
40.0
60.0
80.0
100.0
120.0
140.0
160.0
Sample Report (continued):
Peak ID Compound Time Mass Found
19 6.66
19:(Time: 6.66) Combine (98:100-(88:89+111:112))
IAA
C
2:DAU 176 ES+
1.3e+004
130.2
118.1
129.6
20.5
40.8
45.2
83.6
90.8
98.7
m/z
20.0
40.0
60.0
80.0
100.0
120.0
140.0
160.0
180.0
Sample Report (continued):
Peak ID Compound Time Mass Found
19 6.93
19:(Time: 6.95) Combine (102:104-(91:92+114:115))
ILA
D
4:DAU 245 ES+
2.3e+004
91.1
41.1
27.9
42.2
56.6
67.0
84.0
86.2
97.2
110.8
130.0
130.8
147.2
172.2
173.9
m/z
20.0
40.0
60.0
80.0
100.0
120.0
140.0
160.0
180.0
200.0
220.0
240.0

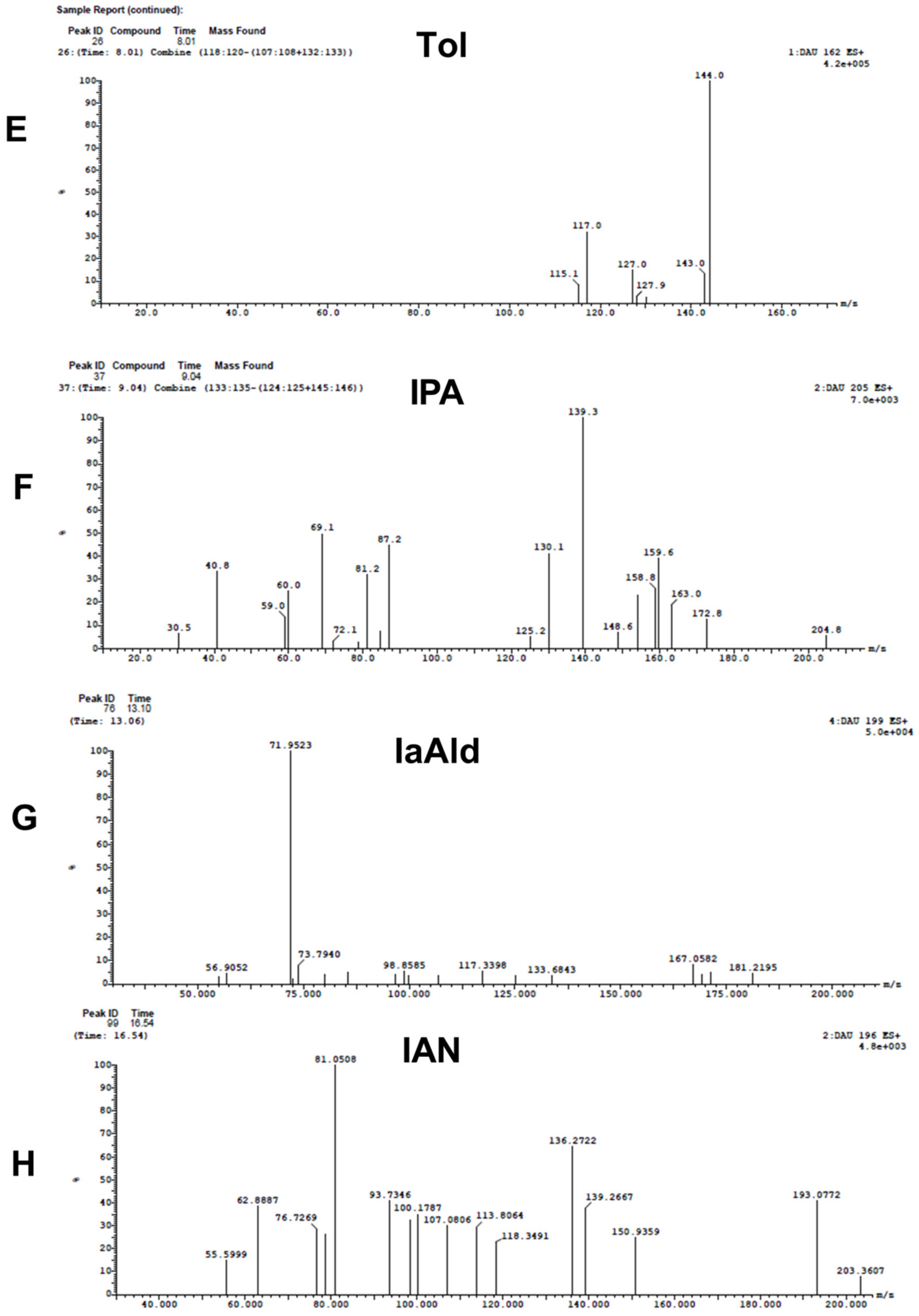

Figure S10. Liquid chromatography–mass spectrometry (LC-MS) fragmentation analysis of targeted indole derivatives in Zch127 at positive mode; (A) 3-methyl indole (skatole), (B) I3CA Indole-3-carboxylic acid, (C) IAA: indole-3-acetic acid, (D) ILA: indole-3-Lactic acid, (E) TOL: indole-3-ethanol, (F) IPA: indole-3-pyruvic acid, (G) IAld: indole-3-acetaldehyde and (H) IAN: indole-3-acetonitrile.

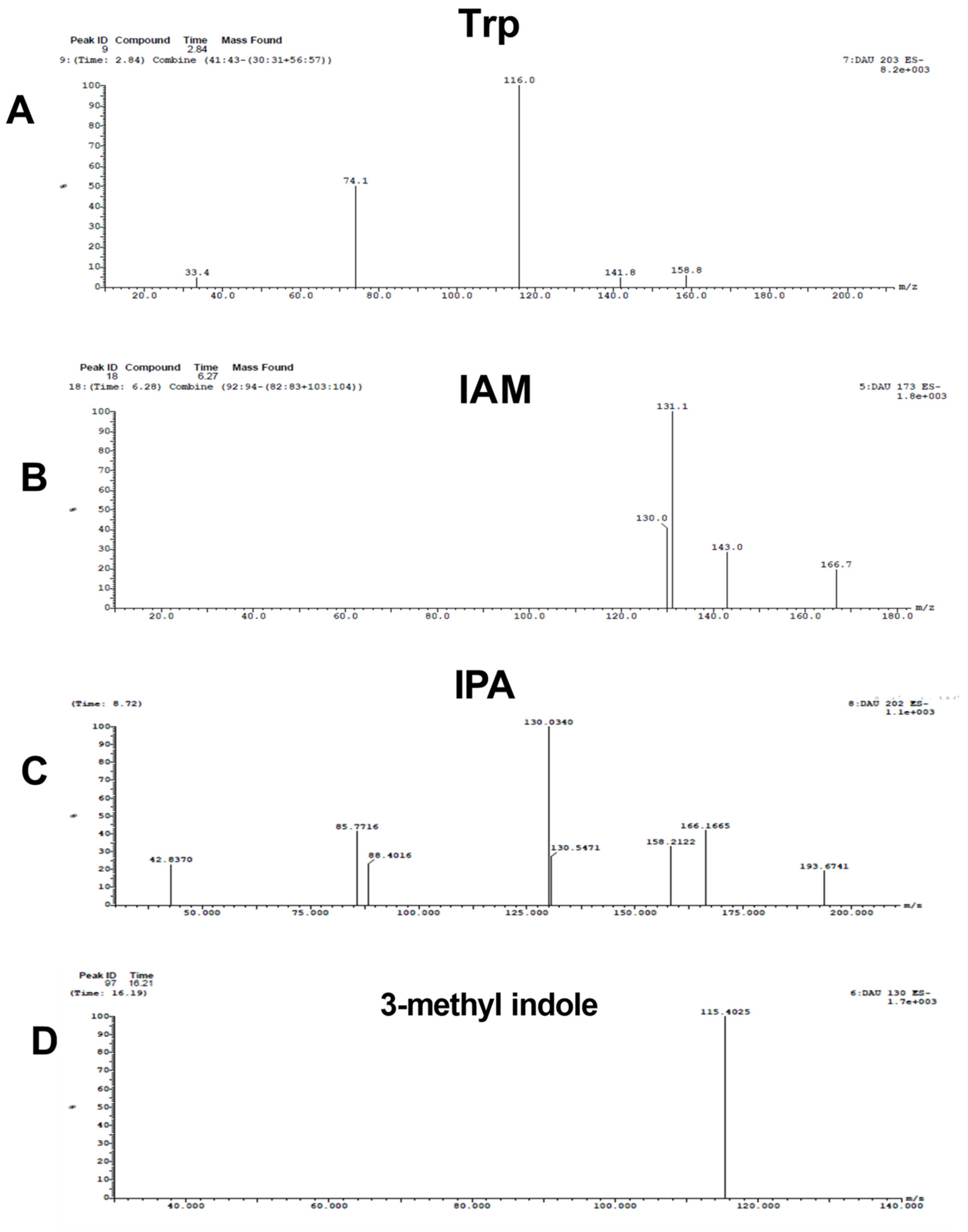

Figure S11. Liquid chromatography–mass spectrometry (LC-MS) fragmentation analysis of the targeted indole derivatives in Zch127 at negative mode, (A) Trp: tryptophan and (B) IAM: indole-3-acetamide, (C) IPA: indole-3-pyruvic acid and (D) 3- methylindole (skatole).
